# Parkinson’s disease progression: Increasing expression of an invariant common core subnetwork

**DOI:** 10.1016/j.nicl.2023.103488

**Published:** 2023-08-22

**Authors:** Phoebe G. Spetsieris, David Eidelberg

**Affiliations:** a Center for Neurosciences, The Feinstein Institutes for Medical Research, Manhasset, NY 11030, United States; b Molecular Medicine and Neurology, Donald and Barbara Zucker School of Medicine at Hofstra/Northwell, Hempstead, NY 11549, United States

**Keywords:** positron emission tomography (PET), Parkinson’s disease (PD), Principal component analysis (PCA), Sparse inverse covariance estimation (SICE), Graphical Lasso (GLASSO), Metabolic connectivity

## Abstract

Notable success has been achieved in the study of neurodegenerative conditions using reduction techniques such as principal component analysis (PCA) and sparse inverse covariance estimation (SICE) in positron emission tomography (PET) data despite their widely differing approach. In a recent study of SICE applied to metabolic scans from Parkinson’s disease (PD) patients, we showed that by using PCA to prespecify disease-related partition layers, we were able to optimize maps of functional metabolic connectivity within the relevant networks. Here, we show the potential of SICE, enhanced by disease-specific subnetwork partitions, to identify key regional hubs and their connections, and track their associations in PD patients with increasing disease duration. This approach enabled the identification of a core zone that included elements of the striatum, pons, cerebellar vermis, and parietal cortex and provided a deeper understanding of progressive changes in their connectivity. This subnetwork constituted a robust invariant disease feature that was unrelated to phenotype. Mean expression levels for this subnetwork increased steadily in a group of 70 PD patients spanning a range of symptom durations between 1 and 21 years. The findings were confirmed in a validation sample of 69 patients with up to 32 years of symptoms. The common core elements represent possible targets for disease modification, while their connections to external regions may be better suited for symptomatic treatment.

## Highlights

• A common core subnet of 11 interconnected regions is identified in cross-sectional PD.
• The core subnet included basal ganglia, pons, vermis, amygdala, and parietal regions.
• This feature was found in over 95% of samples spanning 1 to 21 years disease duration.
• Subnet expression increased with disease duration suggesting a preclinical / prodromal period of approximately 10 years.
• Putamen and globus pallidus exhibited high metabolic activity beginning at the earliest stages.

## Nomenclature

**Acronyms**
AAL: Automated Anatomical Labeling
BCT: Brain Connectivity Toolbox
DC: Degree Centrality
DMN: Default Mode Network
DUR: Duration
EC: Eigenvector Centrality
FDG: Fluorodeoxyglucose Radioactive Tracer
GLASSO: Graphical Lasso
GLASSO/PCA: GLASSO of PC Partition Data
MNI: Montreal Neurological Institute
MATLAB: MathWorks Coding Language
PC: Principal Component
PCA: Principal Component Analysis
PD: Parkinson’s Disease
PDCP: Parkinson’s Disease Cognitive Voxel Pattern
PDRP: Parkinson’s Disease Related Voxel Pattern
PD33_PC1: Parkinson’s Disease Group PD33 ROI Pattern
PET: Positron Emission Tomography
ROI: Region of Interest
ScAnVP: Scan Analysis and Visualization Processor Toolbox
SICE: Sparse Inverse Covariance Estimation
SMA: Supplemental Motor Area
SSM/PCA: Scaled Subprofile Model of Principal Component Analysis
TPR: Topographic Profile Score Rating (SSM Expression Score Evaluation)
subnet: connected subnetwork within a PC partition space
nSparsity: connected network Sparsity
wSparsity: whole brain network Sparsity

## 1 Introduction

Many neurodegenerative conditions manifest distinctive patterns of network connectivity whose underlying etiology and functional associations are poorly understood (Bassett and Sporns, 2017). Diverse methodologies have risen that provide information regarding functional or structural aspects of these associations. Machine learning-based biomarkers have been introduced with some success but the results are not necessarily understandable in terms of disease mechanisms (Katako et al., 2018, Peña-Nogales et al., 2019). Increasing interest has also arisen in multivariate approaches to map changes in metabolic connectivity in disease states (Carli et al., 2021, Hahn et al., 2018, Sala et al., 2017, Yakushev et al., 2017). In Parkinson’s disease (PD) (Obeso et al., 2017, Politis, 2014), principal component analysis (PCA) (Jollife and Cadima, 2016) has been applied to positron emission tomography (PET) metabolic group image data both regionally (Alexander and Moeller, 1994, Eidelberg et al., 1994, Moeller and Strother, 1991) and in voxel-based analysis (Eidelberg, 2009, Spetsieris and Eidelberg, 2011) to identify orthogonal overlapping PC partition layers of the data that reflect specific spatial covariance patterns associated with the disease. These patterns can be distinguished from those associated with other major sources of variance such as atypical parkinsonian conditions, normal network processes including aging, outliers, or noise (Meyer et al., 2017, Schindlbeck and Eidelberg, 2018, Spetsieris et al., 2009, Spetsieris et al., 2015, Spetsieris and Eidelberg, 2011, Tang et al., 2010b). Thus, PCA enables the reduction of the group data into a single pattern distributed over the whole brain, represented by a PC (or linear combination of PCs) and a set of expression scores (subject specific scalars) for the pattern. This approach previously applied to a combined group of healthy subjects and a cross-section of PD patients revealed a robust metabolic imaging PC biomarker (PD-related metabolic pattern; PDRP) indicating higher metabolic activity in the basal ganglia, amygdala, pons and cerebellum and relatively lower activity in parietal and occipital areas (Eidelberg, 2009, Ma et al., 2007, Schindlbeck and Eidelberg, 2018). PDRP subject scores distinguish patients from controls with high reproducibility as has been demonstrated repeatedly in independent populations (Meles et al., 2020, Rus et al., 2020, Schindlbeck and Eidelberg, 2018). By applying graph theoretical analysis (Göttlich et al., 2013, Rubinov and Sporns, 2010) to the whole brain absolute regional correlation matrix of PD and healthy control data, it was shown that a unique regional topographic network relationship existed indicating increased functional metabolic connectivity and clustering within PDRP areas with high region weights compared to other areas (Ko et al., 2018). High correlation of the graphical regional eigenvector centrality with absolute PDRP regional vector weights, as well as characteristic differences in graph theoretical measures of small worldness, were observed over a wide range of graph density values.

In another approach, the Graphical Lasso (GLASSO) algorithm for sparse inverse covariance estimation (SICE) has been applied to neuroimaging data to find the most robust graphical connections of the inverse covariance (precision) matrix without confounding multivariate dependencies. These connections, represented by corresponding partial correlation coefficients, were obtained by penalized reduction of the predicted Gaussian probability (Huang et al., 2010, Ortiz et al., 2015, Sala et al., 2017, Titov et al., 2017). Thus, GLASSO reduces the whole brain data to a network representation of only the strongest direct connections that are independent of third party associations regardless of their source. GLASSO has often been used to compare the connectivity parameters of PD and other condition data to that of healthy control data in diverse pre-determined regions that assume some prior knowledge of the disease topography. Although this approach validates much of the known information regarding the modulation of PD regional connectivity by the disease, it does not provide an integrated picture of disease network inter-associations. Furthermore, in sparse representations of whole brain data, weaker disease-related connections are often obscured by more robust normal connections that are especially prevalent at early disease stages. In view of these concerns, we recently introduced GLASSO/PCA to derive network structure using a data driven approach (Spetsieris et al., 2018, Spetsieris and Eidelberg, 2021). This dual process, i.e., GLASSO of PC partition data, provides a robust model of functional connectivity in specific subnetworks that are more representative of the disease pathways. An advantage of SICE based methods is that major connections are derived with high precision even for small data samples (Yakushev et al., 2017). Thus, we demonstrated (Spetsieris and Eidelberg, 2021) that the application of GLASSO to disease-related partitions of the PD data enabled a more focused evaluation of brain organization in the PDRP space, as well as visualization of the specific connections linking component nodes within the relevant layers.

To observe connectivity associations at different progressive stages of the disease, we apply this dual GLASSO/PCA methodology to cross-sectional imaging data from independent groups of PD patients with sequential increases in symptom duration ranging from 1 to 21 years. We use our new graph theoretical approach to better characterize the changes in metabolic connectivity that underlie PD as the disease advances. Although the phenotypic expression of PD is highly variable (Obeso et al., 2017, Selikhova et al., 2009), there are essential features that define the disorder throughout its course.

We attempted to identify common PD network characteristics intrinsic to the disease process without regard to clinical phenotype or quantitative disability ratings. While a reproducible disease-specific network topography has been established for PD using metabolic PET as well as resting-state fMRI (Rommal et al., 2021, Schindlbeck et al., 2021, Schindlbeck and Eidelberg, 2018, Vo et al., 2017), the identification and characterization of invariant core elements has been somewhat limited (Ko et al., 2018, Rommal et al., 2021, Schindlbeck et al., 2020). Here, to isolate such a subnetwork, we used our totally data-driven approach combining PCA and GLASSO in contrast to GLASSO whole brain or PCA analysis alone, to examine the prevalent network characteristics that were common within all cross-sectional groups of patients, and determined whether there were broad aspects that were present across the 20 year range of duration, and whether others were present that were specific to early, middle or later stages of illness. We note, however, that this general approach can be applied to study specific clinical manifestations of PD such as akinesia-rigidity, tremor, gait disturbance, or cognitive dysfunction. For this purpose, standardized rating scales or cognitive test performance may be used for stratification.

In this study, we identified a distinct common core subnetwork of interconnected regions that form a robust imaging feature of PD, which in retrospective assessment was present from the earliest clinical stages of the disease to extended stages of disease duration. Additional connections within each group are assumed to relate to disease stage and/or phenotype. Mean expression of the subnetwork increased steadily with advancing disease, likely beginning in the prodromal period (Hawkes et al., 2010, Holtbernd et al., 2014, Tang et al., 2010a). Results were validated in a large cross-sectional group and in two independent longitudinal groups of patients. The findings point to potential targets for therapeutic interventions in phenotypically diverse patients and possibly also for treatment of specific clinical symptoms.

## 2 Methodology overview

Methodology is discussed in detail in Methods below. The original group data were analyzed using both whole brain regional SSM/PCA and whole brain GLASSO to examine the relationship between these two contrasting dimensionality reduction methods. Combined GLASSO/PCA analysis, described below, was then used to focus the sparse graphical analysis on the derived disease-specific PC partitions (PD subnets) of the data. Graph theoretical parameters were determined separately for both sparse whole brain and PD subnet connectivity matrices in progressive bootstrap data. Bootstrap analysis was also performed in each patient group to determine the most robust functional metabolic connections that were visualized in 3D representations for the whole brain or the selected PC partition layer of each group and in composite group data. A schematic representation of the overall processing scheme is presented in supporting Fig. S1. A major objective of this study is to demonstrate the advantage of the proposed subnetwork layer analysis over traditional whole brain GLASSO analysis in determining underlying connectivity associations in PD and other conditions. It is assumed that connections derived in the sparse whole brain partition layers of the disease discriminative PC of each group would be more disease relevant. Thus, a disease-invariant common core subnetwork was derived by composite bootstrap assessment of the most prominent connections in the partition layers. Specifically, we evaluated the number of connections that are consistently prevalent across the span of all the PD groups to determine a signature core subnetwork that characterizes PD over the entire cross-sectional population (see below under “Common Core Subnetwork”). Expression levels for the common core subnetwork were tracked over the extensive course of the disease and extrapolated into the prodromal phase. Mean subnetwork expression in each group was tracked in the cross-sectional data as well as in a large group of independent cross-sectional validation data and in prospective longitudinal data in cohorts with early and advanced disease (see below under “Biomarker Validation”).

## 3 Results

### 3.1 Whole brain SSM/PCA

**Fig. 1:**
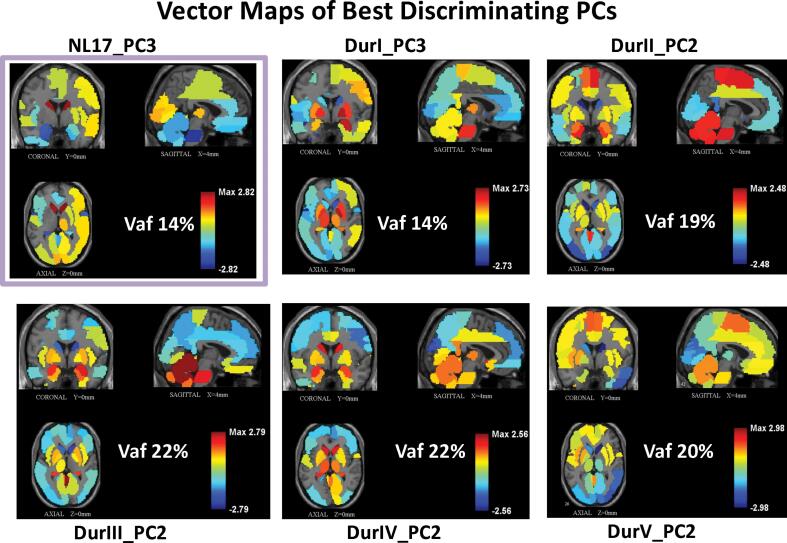
Vector Maps of Best Discriminating PCs. Orthogonal views of regional vector maps are shown over a structural MRI background. The most disease relevant PC in each PD group (Dur I to V) is based on differences in subject score values for the pattern in its derivation group compared to matched healthy subject scores in *t*-test assessment (see text) but discrimination is also significant in the other PD groups (see Fig. 2). NL17_PC3 (*upper left*) scores also discriminated patients vs. controls in all five groups.

**Fig. 2:**
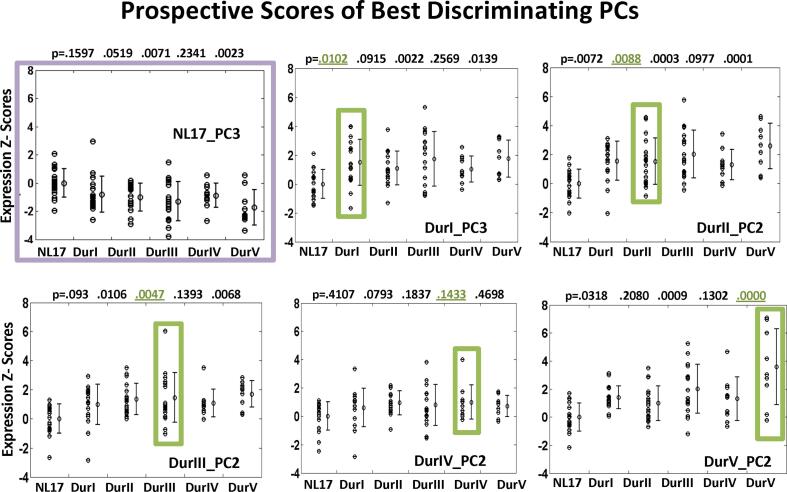
Prospective Scores of Best Discriminating PCs. Scatter plots of repeated prospective *t*-test score assessment of the most disease relevant PC regional vector (Fig. 1), shown in green rectangles for the derivation group are further evaluated in each group DurI, II, III, IV and V. Vector map TPR scores were Z-scored with respect to NL17 subject scores. Each PD group was compared to healthy control subjects using post-hoc Dunnett’s tests; corresponding p-value are given at the top each column. These PCs also discriminated at least two other PD groups from the NL17 subjects with the exception of DurIV_PC2 that showed a trend in one other group. NL17_PC3 discrimination (*upper left*) was shown here for comparison. In contrast to the other patterns, the normal pattern indicated overall decreasing mean expression with progressing PD duration. (For interpretation of the references to color in this figure legend, the reader is referred to the web version of this article.)

SSM/PCA regional whole brain analysis was performed separately for each of the five independent PD groups with increasing symptom duration (DurI-DurV; see Table 1), and for the age- and gender-matched healthy control group (NL17). We evaluated SSM PC expression scores to determine the best discriminating PC in each group. We found that scores for a single PC (vector maps depicted in Fig. 1), corresponding to PC3 in DurI and NL17 and PC2 in the other PD groups, significantly discriminated patients from control subjects (p = 0.0024, 0.0021, 0.0048, 0.0298, 0.0038 for DurI-DurV, respectively compared singly in their derivation group to healthy control subjects in comparisons; Student’s *t*-tests; see Fig. 2 *green rectangles*). In prospective evaluation in each of the other groups, values were corrected for multiple comparisons with respect to the same control group, in separate Dunnett's tests (Pokala, 2012), (p=0.0102, 0.0088, 0.0047, 0.1433, 0.0000, see Fig. 2 *top of panels, green rectangles*; one-way ANOVA, Dunnett’s tests). Discrimination is expected to be higher in the derivation group but we noted expression values for these PD-related PCs (DurI_PC3, DurII_PC2, DurIII_PC2, DurV_PC2) also discriminated other patient groups from healthy controls (Fig. 2), often at higher significance levels (lower p-values) even when corrected for multiple group comparisons. Although not necessitated for the analysis, this provided extended validation for these patterns, with the exception of DurIV_PC2 for which discrimination did not reach significance in any group in multiple comparisons. However, its discriminative capacity was verified when more appropriately tested prospectively in a large group of 33 PD patients and 33 matched controls (p = 6.6e-007, Student’s *t*-test). Moreover, all of the PD Dur patterns exhibited relative increases in activity as part of the network (positive region weights) in the putamen, globus pallidus, cerebellum, and pons, accompanied by reductions (negative region weights) in parietal and occipital association regions. This topography was consistent with that of the previously validated PDRP voxel-based pattern (Schindlbeck and Eidelberg, 2018, Spetsieris and Eidelberg, 2011, Ma et al., 2007) and the related PD33_PC1 regional pattern (Ko et al., 2018, Spetsieris et al., 2018, Spetsieris and Eidelberg, 2021). It should be noted that the PDRP was never directly involved in the identification of the common core subnet. Significant correlations of the regional disease patterns for each PD group with PD33_PC1 were observed (r = 0.36, 0.66, 0.74, 0.63, 0.70; p < 0.001, Pearson’s correlations), while the healthy pattern correlated negatively with the disease pattern (r = -0.65, p < 0.001). In contrast to the other patterns, DurIV_PC2 exhibited positive region weights in the caudate nucleus. The DurIV_PC2 pattern (Fig. S2) correlated strongly (r ∼ 0.62) with voxel weights on the PD cognition-related pattern (PDCP), a disease topography associated with cognitive decline (Mattis et al., 2016, Schindlbeck et al., 2021, Schindlbeck and Eidelberg, 2018). The primary pattern in group IV, DurIV_PC1 was not significantly discriminative for group DurIV but significantly separated the two adjacent patient groups (DurIII and DurV) from healthy control (NL17) values (p ∼ 0.05 and p ∼ 0.04, respectively, Dunnett’s test; Fig. S2). Its distinct topography characterized by reduced activity in caudate, parietal, and occipital regions and relatively increased activity in the frontal cortex, suggested the appearance of an additional disease network in later stage patients related to the DMN (Fig. S2). Both DurIV_PC1 and marginally DurIV_PC2 patterns appear to be incorporated in the DurV_PC2 pattern based on regional correlation values (r = 0.63 and r = 0.15, respectively; Fig. S2).

**Table 1:** Group Demographics.

Group	[n]	Disease Duration^a^ (years)	Gender (M/F)	Age^a^ (years)	UPDRS (motor)^a^ [n]
PD70					
PDI	[17]	[1–3], 1.9 (0.9)	13/4	[45–70], 61.5 (7.4)	[5–29], 15 (8.7) [11]
PDII	[17]	[4–7], 5.7 (1.0)	11/6	[51–72], 60.2 (6.3)	[10–45], 27.3 (9.2) [15]
PDIII	[17]	[8–12], 9.9 (1.6)	13/4	[51–70], 60.9 (7.7)	[15–53], 33.5 (13.5) [16]
PDIV	[10]	[13–16], 13.9 (1.2)	8/2	[51–68], 60.4 (5.2)	[13–56], 31 (13.7) [9]
PDV	[9]	[17–21], 18.6 (1.4)	6/3	[50–70], 62.2 (7.3)	[10–69], 38.3 (18.7) [9]
					
NL17	[17]	N/A	12/5	[46–71], 61.6 (8.5)	N/A

a [Range], mean (SD).

### 3.2 Whole brain GLASSO versus GLASSO/PCA

To derive the prominent connectivity of the original data set, we performed a preliminary study where we applied GLASSO to the data from the whole brain, as well as to the subnet partition data associated with the disease related PC in each derivation group (Fig. S1A). The GLASSO penalty parameter was varied and the sparsity and EC of the connected subnetwork was evaluated in each case. For the disease-related subnet partitions, strongly connected subnetworks involving all nodes could not be obtained. Sparsity was increased to remove noisy edges and ensure that the EC of the adjacency matrix correlated well with the corresponding absolute disease PC region weights. Selected cases typically included highly weighted disease relevant nodes whose edges presumably represent the connectivity of the disease network. For whole brain, we increased the penalty parameter to determine the maximum sparsity that maintained a strongly connected network so that the prominent nodes of the PC based analysis are included and connected. We found that the maximum whole brain sparsity that ensured strong connectivity in whole brain was approximately 85% in all groups but the EC of the connected network did not necessarily correlate with the disease PC vector. For partition data the smaller number of connected edges (approximately 350 compared to about 650 for whole brain) in most cases corresponded to a similar level of network sparsity of about 85% as for whole brain. Similar network sparsity is often considered a criterion for comparison of networks with a differing number of edges. Because of left out nodes and edges, the derivation subnets had a higher wSparsity value of approximately 92% over all nodes. Results for levels near 92% wSparsity in whole brain GLASSO analysis were not included because of missing prominent disease nodes whose connectivity and graph theoretic properties could not be evaluated for comparison to that of the subnet analysis. Similarly, many of the bootstrap sample connected subnets did not include all prominent nodes at higher 92% wSparsity levels. We therefore evaluated 85% wSparsity levels in addition to 92% wSparsity levels for partition subnets in bootstrap analysis.

**Fig. 3:**
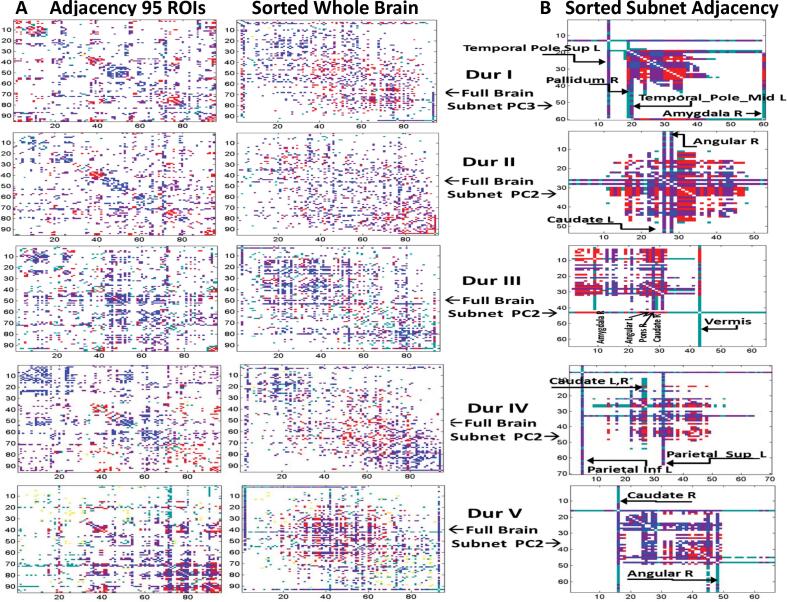
Adjacency Matrices of Whole Brain and Subnet Partitions in Fiedler Sorting. (A) Adjacency matrices of the whole brain (95 ROI) connected networks at maximum sparsity for the derivation samples in progressive groups DurI to V. *Left (1st column):* indexed by unsorted regions 1 to 95. *Right (2nd column):* re-sorted by the ROI index of increasing values of the Fiedler vector elements. (B) Adjacency matrix of the disease relevant PC sparse connected subnet of each group displayed in re-sorted ROI index order corresponding to increasing magnitude of the subnetwork Fiedler vector elements. Connections of high EC nodes (EC > 1 SD) are depicted as red, blue or magenta points. Red points correspond to connections of positive regions, blue with negative and magenta with positive to negative regions of the derived disease PC. Cyan points indicate high EC with low EC node connections and a minor number of yellow points found in whole brain indicate low EC connections. Hub nodes with high degree centrality (*lines*) are noted. The corresponding number of edges, number of ROIs and sparsity in terms of whole brain (wSparsity) and within the subnet configuration (nSparsity) are recorded in the text. (For interpretation of the references to color in this figure legend, the reader is referred to the web version of this article.)

The adjacency matrices for each of these groups are displayed in Fig. 3 which also depicts their Fiedler re-sorted connections (see Methods) in terms of positive and negative nodal values for the corresponding disease PC vector regions as described below. The prominent (core) nodes that are included in all of these subnet configurations are highlighted in Table 2 where these high weighted hub nodes for which (EC ≥ 1) are indicated by asterisks in each group (Table 2, columns I to V).

**Table 2:** Subnetwork Hub Nodes of the 95 Region Atlas.





High ranking subnet nodes for each category for the 95 evaluated atlas nodes are listed here. Subnet hub nodes for which EC > 1 SD in the derivation sample GLASSO/PCA analyses (columns DurI to DurV) or high ranking nodes in composite (I to V) or (I to III) bootstrap core networks at 85 ± 5% or 92 ± 2 % sparsity range assessments are indicated by an asterisk. Magenta highlighted nodes represent nodes that appear in at least four of the progressive Dur derivation groups. Bootstrap at 85% (column 6) and 92% (column 7) high ranking core subnet hub nodes (see text) are depicted by cyan and yellow for composite I to V groups (see also Fig. 6A). At 92%, only 5 yellow highlighted nodes indicated by the number symbol (#) were ranked in the top 5% while the remaining nodes were prevalent within the upper 20% sparsity range. The far right column 8 depicts the upper 5% nodes in composite I to III bootstrap assessment within both ranges highlighted in green. The lower 85% range included 24 nodes including 11 indicated by the number symbol (#) that remained prevalent in the top 5% of the 92% range (see also Fig. 6B).

### 3.3 Laplacian spectral decomposition

For both the full brain and the subnet analyses derivation samples, connectivity relationships in the sparse disease-related topography were delineated using the graph Laplacian matrix (Spetsieris and Eidelberg, 2021). The adjacency matrix was re-sorted by magnitude according to the ascending nodal order of the eigenvector corresponding to the first (smallest) non-zero eigenvalue of the graph Laplacian matrix (i.e., the Fiedler vector; see Methods for details). The adjacency matrices of the derivation group samples and their re-sorted representations are shown in Fig. 3A, B. These displays highlight nodal associations along the graph: whole brain sparse representations of adjacency are composed of diffuse modules of positive and negative high EC node connections (Fig. 3, *middle column*) which form distinct dense clusters in the subnet configurations (Fig. 3, *right*); low EC nodes are connected peripherally. The point elements of these matrices (edges) were colored based on whether they connected high EC (EC ≥ 1 SD) hub nodes and whether the polarity of the two nodes within the group-related disease PC vector was positive or negative. Thus, connections linking high EC positive nodes are shown as red, those between high EC negative nodes are shown in blue; connections linking positive and negative high EC nodes are shown in magenta. Connections linking high EC to low EC nodes are depicted as cyan. Connections between low EC nodes are depicted in yellow. Of note, low EC node connections were infrequent in the sparse whole brain representations, and were totally absent in the sparse subnetworks.

In the whole brain matrices (Fig. 3A, *right*), we noticed modest modularity without clear-cut graph partitioning. Red and blue edges tended to group into diffuse separate modules. That said, subnetwork spectral evaluation (Fig. 3B) revealed distinct core clustering (dense red, blue and magenta areas) between high EC nodes (hubs). Certain high EC hub nodes formed discrete connections (*solid lines*) with all the nodes within the subnet (labeled solid lines in B). The blue, red and magenta areas of these solid lines correspond to connections with the core high EC nodes within the cluster and the cyan areas correspond to separate peripheral connections with weaker nodes. The large white subnet space with zero adjacency results from limited low EC connections to one or two central hubs and the visualized separation of high and low EC connections into separate spaces of the adjacency matrix following the sorting procedure.

The identity of the high EC hub nodes of the subnetwork configurations of groups I to V are noted in Table 2, revealing certain regions as distinct common components of the core in each of the progressive group clusters. Thirteen nodes including the pons, putamen, caudate, angular gyrus, and inferior parietal regions bilaterally, as well as the vermis and right amygdala and hippocampus, were identified as common core hubs, as defined by EC > 1 SD in at least four of the Dur PD derivation samples analyzed with subnet GLASSO/PCA. For each sample, these regions were present in the clusters depicted in Fig. 3 (*right*) and highlighted in magenta in Table 2, columns I to V. Eleven of these common core subnet hub nodes were also identified as the nodes that formed the top 5% (95% or more instances) of composite connections in the 85 ± 5% bootstrap analysis of the combined PD DurI to V group (highlighted in cyan in Table 2 (column 6)). The 13 nodes also included the 12 highlighted in yellow in Table 2 (column 7), which were identified as forming the top 20% of the composite connections in the 92 ± 2% bootstrap analysis.

### 3.4 Early stage PD

**Fig. 4:**
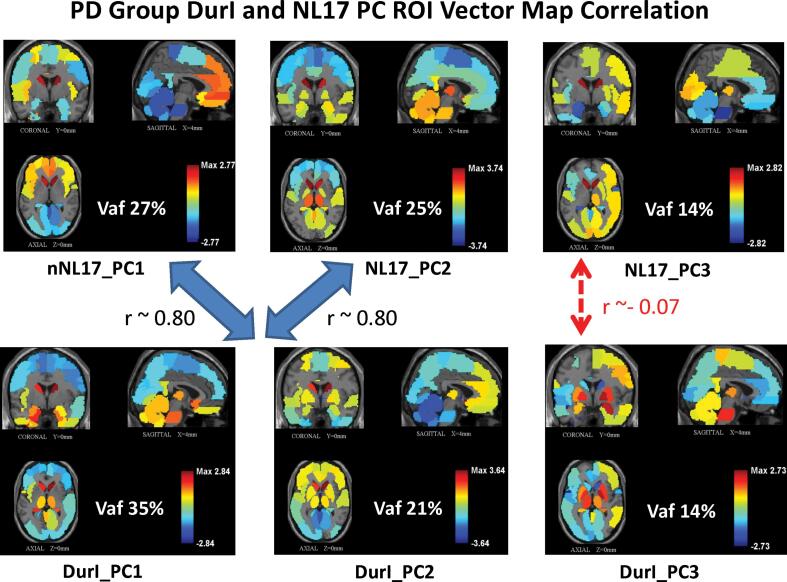
PD Group DurI and NL17 PC ROI Vector Map Correlation. Orthogonal views of regional vector maps are presented for the three primary PD PCs in group DurI (*bottom*) and the healthy group NL17 (*top*) for comparison. High switched order correlation (|r|∼0.8) is apparent for the corresponding first two PCs in each group whose subject scores did not discriminate between patients and healthy controls but corresponding PC3 patterns that showed significant discrimination did not correlate (|r|∼0.07).

We compared the earliest PD group DurI (1-3yrs) to the healthy group NL17 (Fig. 4) based on their first three principal components. Together, the first two PCs accounted for similar amounts of the total variance (56% and 52%) in the patient and control groups, respectively. PC1 and PC2 in DurI resembled their counterparts in NL17, but in reverse order: the correlation of their corresponding vector maps (PC1 of DurI with PC2 of NL17 and vice-versa) was approximately 64% (|r|≈0.8). In both groups, each of the two PC patterns exhibited moderate correlation (|r| ranged from 0.5 to 0.64) with the metabolic default mode network (DMN) that was previously identified in healthy subjects (Spetsieris et al., 2015). However, the third PCs, each accounting for about 14% of the variance, were not correlated with each other (r^2^ ∼ 0.005, |r|≈0.07, Fig. 4, *right*) or with the DMN (r^2^ < 0.04). In contrast to NL17_PC3 that exhibited high positive activity in the caudate and high negative values in vermis and pons (Fig. 4, *top right),* the DurI_PC3 pattern (Fig. 4*, bottom right*) exhibited high positive values in the cerebellar vermis and pons, basal ganglia, thalamus and motor areas. Surprisingly, negative NL17_PC3 patient scores significantly discriminated each of the 5 PD Duration groups I to V from NL17 group scores (p = 0.05, 0.006, 0.004, 0.03, 0.008; Student’s *t*-tests) for the respective comparisons and (p = 0.16, 0.05, 0.007, 0.234, 0.002; one-way ANOVA, Dunnett’s test) corrected for multiple comparison**,** suggesting that this healthy network was directly affected by the disease even at the earliest stage (Fig. 2, *top, left*). Interestingly, NL17_PC3 correlated negatively to a variable degree with the discriminative PD Dur patterns (|r|≈0.07, 0.58, 0.62, 0.41, 0.54, p < 0.001 for PD DurI to V; Pearson's correlations). This likely reflects region weight values of opposite sign in extrastriatal areas such as pons and vermis at all disease stages.

### 3.5 Bootstrap analysis

**Fig. 5:**
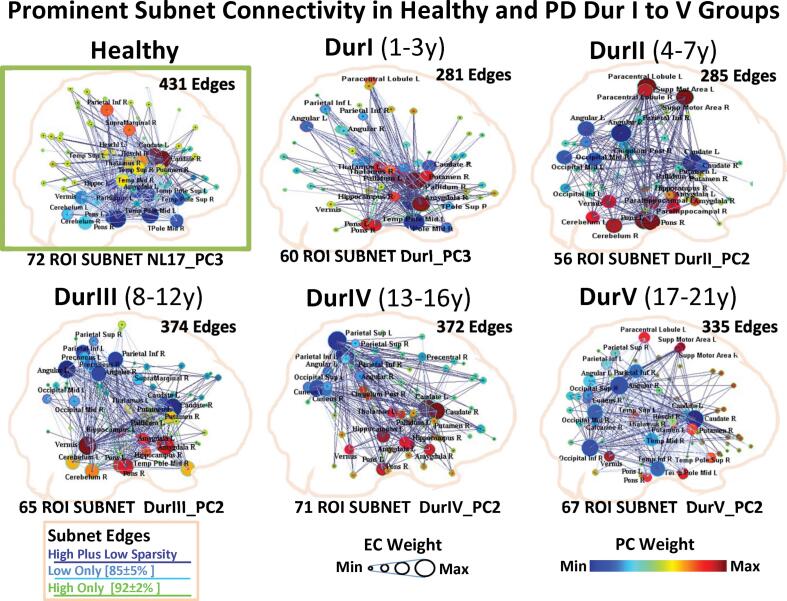
Prominent Subnet Connectivity in Healthy and PD DurI to V Groups. Common edges were derived at whole brain sparsity 85 ± 5% for 100 bootstrap samples in each group’s subnet data and were rederived at 92 ± 2% sparsity. Node diameter corresponds to the EC of the subnet of strongly connected common sample connections for that group at 85 ± 5% sparsity. Color corresponds to signed PC region weight of the corresponding derivation group. (Correlation of the EC vector with the corresponding absolute PC region weights was r^2^ = 0.96, 0.98, 0.75, 0.96, 0.67, p < 0.0001, for DurI to V, respectively at 85 ± 5% sparsity.) The number of connected edges was, respectively, (281, 285, 374, 322, 335) for an effective nSparsity of (84, 83, 82, 87, 85), mean ∼ 84%. At 92 ± 2% sparsity, correlation of the EC vector with the corresponding absolute PC region weights for PD was r^2^ = 0.96, 0.97, 0.95, 0.97, 0.96, p < 0.0001, for DurI to V, respectively. The number of connected edges was, respectively, (227, 251, 243, 260, 200) for an effective nSparsity of (87, 82, 86, 88, 91), mean ∼ 87%. For NL17_PC3, r^2^ = 0.89, edges = 236, effective nSparsity = 87%. The common edges for the two sparsity ranges are depicted in dark blue. Edges that were prominent only at lower sparsity are shown in lighter blue and a few edges that were only prominent at high sparsity are shown in green. (For interpretation of the references to color in this figure legend, the reader is referred to the web version of this article.)

To derive the most prominent connections at different disease durations, we performed GLASSO analysis of the whole brain and the disease-specific PC partition data in over 2000 cases to find 100 bootstrap samples with viable solutions for each data set and category of the DurI-V and NL17 groups across the wSparsity ranges of 85 ± 5% and 92 ± 2% for partition data and 85 ± 5% for whole brain analysis (see Methods). The weight of the connections was determined from the relative number of occurrences of each connection in the individual categories. A 3D representation of prominent subnet connections that occurred in all samples for each PD group and for the healthy NL17_PC3 partition is presented in Fig. 5 (joint sparsity range). Node diameter corresponds to the weights of the common EC vector nodes in each group at the lower 85 ± 5% wSparsity range. In Fig. 5 connections that were prominent only at lower sparsity are shown in lighter blue color while the dark blue edges are prominent at both sparsity levels. Only a handful of connections, shown in green in Fig. 5, are prominent only at high sparsity. Thus, the composite configuration of the higher 92 ± 2% wSparsity range is essentially incorporated in the composite lower 85 ± 5% wSparsity representation which also includes additional weaker connections. The mean effective subnet sparsity for prominent nodal connections was 84% for 85 ± 5% wSparsity and 87% for the 92 ± 2% wSparsity range (see Fig. 5 legend). Correlation of the bootstrap group-specific common subnet EC vector with the corresponding absolute PC region weights for 85 ± 5% wSparsity was r^2^ = 0.96, 0.98, 0.75, 0.96, 0.67, p < 0.0001 for DurI to V respectively, but higher (r^2^ > 0.95, in all groups) in the 92 ± 2% wSparsity range because of the elimination of the more weakly connected nodes (Fig. 5).

### 3.6 Healthy and early stage PD

In bootstrap analysis, the graph for PC3 in the healthy control group (Fig. 5, Healthy) exhibits salient EC weights in the pons, caudate nucleus, and temporal cortex, which are also present in patients throughout the course of their disease (Fig. 5, DurI to V). That said, the sign of the corresponding PC weights on these regions differed in the healthy and PD groups. Striking differences between early PD and normal were seen for connections involving the globus pallidus (diminished in the healthy graph) and amygdala, and involving the parietal cortex at high sparsitiy (Fig. 5, Healthy and DurI). The light colored nodes (putamen, cerebellum, etc.) of the normal graph (Fig. 5, Healthy) have analogous low EC at high sparsity because of the elimination of many low weight connections evident at low sparsity (*light blue lines*). Thus, for the healthy configuration, the putamen and thalamus nodes appear large in the low sparsity representation (Fig. 5, Healthy) but are diminished at high sparsity. However, for the early PD representation, these nodes are larger and appear in tight clusters that are maintained at high sparsity (Fig. 5, DurI). By contrast, the caudate has a higher EC in the healthy group that is maintained at high sparsity, whereas the pallidum, paracentral lobule and angular gyrus, which are prominent in the PD DurI representations, are absent in the healthy representations.

### 3.7 Mid stage PD

At the intermediate stages of PD, exemplified by DurII (4 to 7 years) and III (8 to 12 years), we noted a relative increase in both negative and positive extrastriatal regions affected by the disease as noted in 3D representations of the prominent connections seen on subnet bootstrap analysis (Fig. 5, DurII-III). In both groups, we observed increased connections of the positive cerebellar vermis and hemispheres, and the pons with the rest of the brain, as well as those involving negative cortical areas bilaterally in the angular gyrus, inferior parietal cortex, and occipital lobes, which overshadow persistent connectivity changes involving the striatum. Other notable changes appear to be group specific. Increased relative activation is noted bilaterally in the paracentral lobule and supplemental motor area (SMA) in the initial mid-stage group (DurII) which are absent at the later intermediate stage (Dur III). In further comparison, the DurIII group shows an increase in connections involving slightly negative frontal areas. This is in broad agreement with the gradual progression of the disease following the appearance of clinical symptoms described by Braak and others (Braak et al., 2003, Hawkes et al., 2010).

### 3.8 Late stage PD

In advanced PD (Fig. 5, DurIV, V), we noted a topographical reorganization of functional associations as noted in derivation data (Fig. S2). The DurIV_PC2 disease pattern showed the greatest discrimination from healthy subjects in comparison (p = 0.03, Student’s *t*-test) although not in multiple comparisons (p = 0.14, Dunnett’s test) (Fig. 2, Fig. S2E). While subject scores for the primary pattern (DurIV_PC1) did not differ for DurIV patients compared to healthy control subjects (p = 0.19; Student’s *t*-test and p = 0.43; Dunnett’s test), these values reached significance or marginal significance corrected for multiple comparison when computed in PD patients in the immediately earlier (DurIII) and later (DurV) PD groups compared to corresponding healthy control scores (p = 0.04 and p = 0.01, respectively, for Student’s *t*-tests, and p = 0.05 and p = 0.04, respectively, for the post-hoc Dunnett’s test) (Fig. S2D). The PC2 disease pattern of the DurV group contained element s of DurIV_PC1 and PC2, but the correlation with the former pattern (r ∼ 0.63) was dominant (Fig. S2C). Thus, the sparse whole brain connectivity of DurIV is influenced by all of its subnet components but elements of the caudate appear to play a different role in the first two subnets appearing red (high positive region weight) in DurIV_PC2 (Fig. S2B) and blue (low negative region weight) in DurIV_PC1 (Fig. S2A). These results suggest a multifunctional role of the caudate in both normal and disease processes in the brain.

The changes in connectivity observed at the later stages of PD (Fig. 5, DurIV, V) are not as well defined as at earlier stages. These findings are consistent with the greater variation in clinical manifestations such as cognitive dysfunction seen in patients with long duration disease. We note that not all DurIV patients express high scores for the selected discriminative network DurIV_PC2 while some exhibit elevations in scores for DurIV_PC1. Network inter-connectivity is reduced in DurIV_PC2 and DurV_PC2 partitions as evidenced by fragmentation of large clusters into distinct modules (Fig. 3). Indeed, whole brain clustering in DurIV and V becomes more apparent as individual core nodes form diverse network associations. Also of note, involvement of healthy networks such as DMN, which was not seen at earlier disease stages, now become evident. This is demonstrated by the significant correlation of DurIV_PC1 with the normal DMN topography (r = 0.43, p < 0.001) (Fig. S2), whereas the more discriminative DurIV_PC2 pattern is more closely correlated with the abnormal PDCP topography (r = 0.62, p < 0.001).

### 3.9 Common core subnetwork

**Fig. 6:**
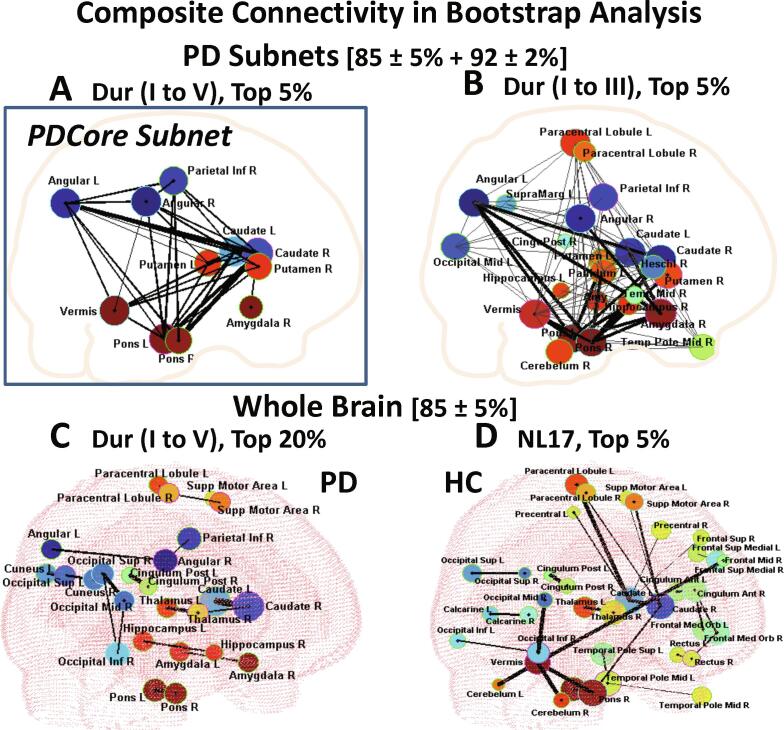
Composite Connectivity in Bootstrap Analysis. Prominent bootstrapped connections in composite PD subnet (A, B) and whole brain PD (C) and healthy group (D) data is shown at 85 ± 5% sparsity. Composite PD connections at 92 ± 2% sparsity are superimposed in the subnet configurations (A, B). The composite adjacency of 100 bootstrapped samples in each group was combined (total 500 PD subnets (A), 300 subnets (B), 500 PD whole brain (C) and 100 healthy samples (D)) separately for each sparsity range. Thus, an additional 500 subnets for (A) and 300 subnets for (B) were assessed at higher sparsity for the final configuration. Node diameter was determined by average bootstrap EC weight of the corresponding whole networks or PD group subnetworks at 85 ± 5% sparsity. Node color was determined by average signed PC region weight of the corresponding discriminating PD group PCs (PC3 for DurI and PC2 for each of DurII to DurV in (A) and (C) and in (D) for comparison. Only the first three Dur group discriminating PCs were averaged to designate node color in (B)). (A) PD subnets top 5% of connection (475 to 500 instances in composite I to V samples) at 85 ± 5% sparsity with superimposed connections (for 475 to 500 additional instances) at 92 ± 2%. Line width (LW) = 1 for upper 5%, LW = 2 for upper 3% and LW = 4 for top value 500. Eleven “common core subnet hub nodes” interconnected by 38 edges were identified as the vermis, right amygdala, right parietal inferior and left/right caudate, putamen, pons and angular gyrus (Table 2, column 6). Only four edges including connections of the right caudate to the left angular gyrus, the left and the right pons and the vermis connection to the left pons were found for upper 5% prevalence at high 92 ± 2% sparsity (Table 2, column 7). (B) Top 5% (285 to 300 instances in composite I to III bootstrap) that are prevalent in the composite first three Dur stages I to III for each sparsity range. At high sparsity, the upper 5% most prevalent edges (thick black lines, LW = 3) consist of 18 connections involving 11 nodes (Table 2, column 8, symbol #) of which 10 are common to the 11 nodes (Table 2, column 6, symbol *) found at the lower sparsity for the composite five Dur groups shown in (A). The 18 high sparsity edges are included in 98 edges involving 24 nodes prevalent in the upper 5% of the lower sparsity range (shown in gray, LW = 1), (Table 2, column 8). Lower sparsity edges include connections of the hippocampus, cerebellum, left pallidum, paracentral and occipital areas (Table 2, column 8) that are absent in the composite I to V common core subnet shown in (A). (C) PD whole brain. Common top 20% of connections (400 to 500 instances in composite groups I to V bootstrap) at 85 ± 5% sparsity. LW = 2 for upper 20%, LW = 4 for upper 10% and LW = 6 for upper 5%. The maximum 496 connections was found for the left and right caudate. (D) Healthy (group NL17) whole brain top 5% of connections (95 to 100 instances) at 85 ± 5% sparsity. LW = 1 for upper 5%, LW = 2 for upper 3% and LW = 4 for top value 100.

To derive the core subnet connectivity that was common to all the PD groups, we first examined the connections that were present in the upper 5% of the composite 500 bootstrap sample submatrices for 85 ± 5% wSparsity (Fig. 6A, Supporting Information Table S1). We found that for 475 to 500 samples, the common connections involved 11 hub nodes interconnected by 38 edges comprising a core subnet that represented <1% of the 4465 possible undirected edges of the whole brain graph. The common core subnet hub nodes, which included the caudate, putamen, pons, cerebellar vermis, and angular gyrus bilaterally, and the right inferior parietal cortex and amygdala, are highlighted in cyan in Table 2, column 6. In all 500 samples the right caudate connected to the left caudate and to the right and left pons, and the left angular gyrus, while the left caudate connected to the left putamen. See Table S1 for list of the most prevalent connections.

For 92 ± 2% wSparsity, only three connections linking the right caudate with the left angular gyrus and the left and right pons were prevalent in 500 samples tested at the high sparsity level (highlighted in Fig. 6A). An additional connection linking to cerebellar vermis and the left pons was prevalent in the upper 5%. At the top 20%, prevalent connections were seen that were very similar to those for the upper 5% at the lower sparsity (Table 2, column 7). Thus, at high sparsity some important disease relevant nodes may be obscured because of their lower connectivity. For comparison, we identified the most prominent connections that are prevalent in the composite early and intermediate (DurI-III) patient groups at both sparsity ranges (Fig. 6B). At high sparsity, the upper 5% most prevalent edges (*thick black lines*, Fig. 6B) consisted of 18 connections involving 11 nodes (Table 2, column 8, symbol #) of which 10 were common to the 11 nodes (Fig. 6A; Table 2, column 6, symbol *) found at the lower sparsity for the full patient sample range (DurI-V). All of the high sparsity connections were also found among the 98 edges (linking 24 nodes) that were prevalent in the upper 5% of the lower sparsity cases observed in the composite subnet of the combined DurI-III group (Fig. 6B). The additional lower sparsity edges (*thin gray lines*, Fig. 6B) include connections of the hippocampus, cerebellum, left pallidum, paracentral and occipital areas that are eliminated in the composite I to V common core subnet because of lower connectivity in the more advanced (Dur IV and V) groups that were included (Fig. 6B, Table 2, column 8).

For the whole brain configuration, fully connected adjacency matrices could not be obtained at the high sample sparsity of 92 ± 2% for PD groups I to V or for the healthy NL17 group. We identified the most prevalent connections in the bootstrap adjacencies of the 85 ± 5% sparse PD whole brain composite assessment, of which the left and right caudate connection was the most reliable (496 out of 500 samples). The top 20% of connections (400 to 500 instances in composite groups I to V; Fig. 6C) are not fully interconnected. Compared to subnet configurations, many of the same nodes are also present in whole brain but with less reliability. Under these circumstances, the connections of primary nodes such as the putamen, vermis and cerebellum do not survive the sparsity threshold. The representation of the healthy whole brain upper 5% of prevalent connections for 100 bootstrap samples (group NL17) are shown in Fig. 6D. For this composite pattern, the caudate, pons, and cerebellum were the most reliable, highlighting their role in normal brain networks. Notably the PD prevalent nodes of putamen, angular gyrus and right inferior parietal were absent from this display while several frontal and other distributed nodes are prevalent only in the healthy configuration. For these nodes, high EC (diameter) does not correspond to deep color tones that signify prevalence in PD.

**Fig. 7:**
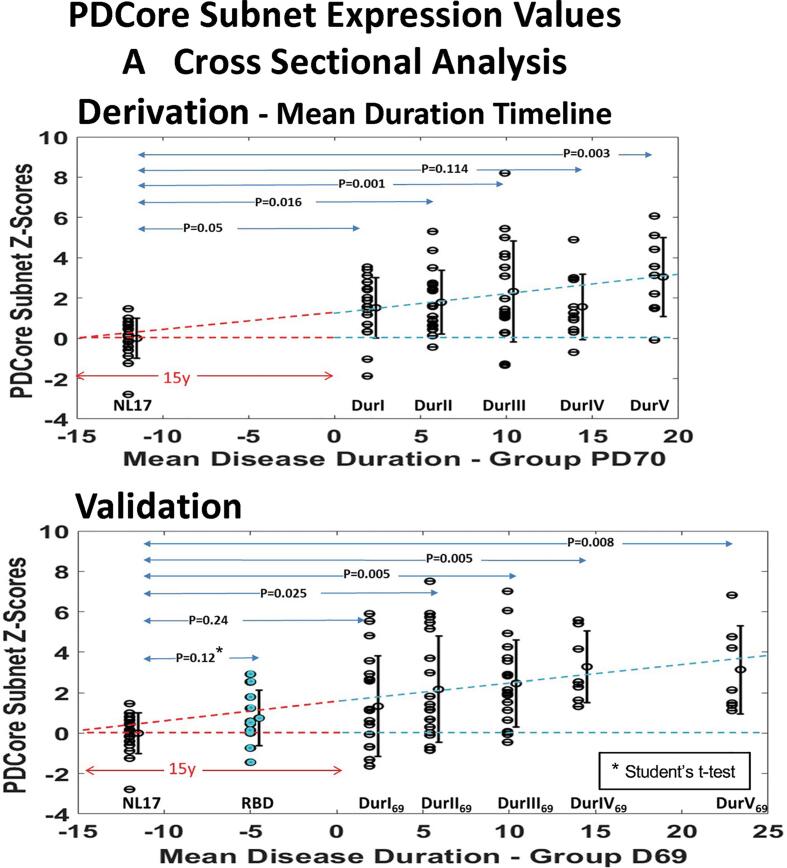

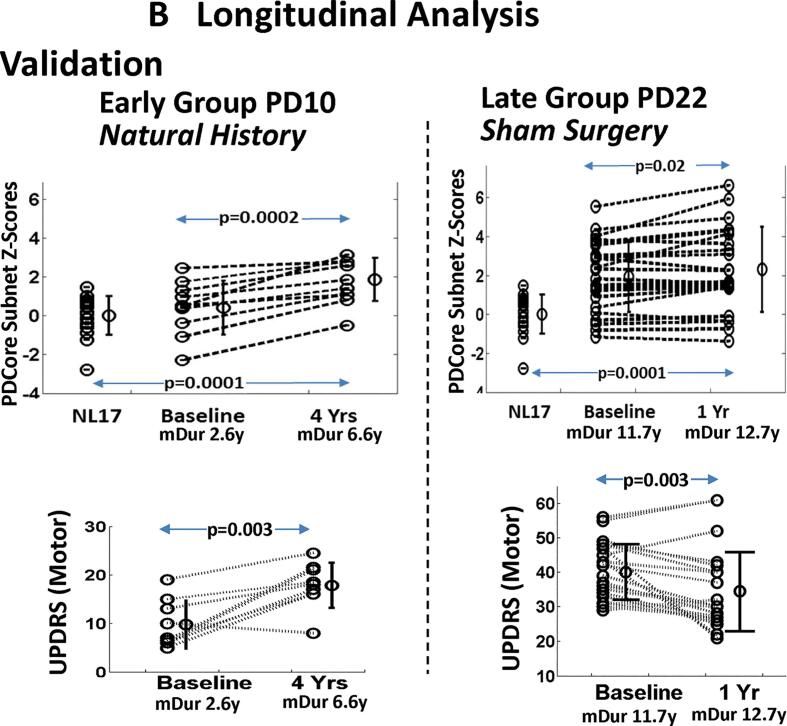
Common Core Subnet Subject Expression. PDCore_subnet expression values were computed in the derivation group (Table 1) and in the independent patient data (Demographics Table S2). (A) Cross Sectional Data: Prospective scores for the individual patients and the predicted timeline for the mean PDCore_subnet expression (*dashed line*) are displayed for the derivation data (Group PD_70_, *top*) and the validation data (Group PD_69_, *bottom*). Differences in PDCore_subnet expression for each of the PD groups (DurI – DurV) compared to NL17 were significant in both data sets (p < 0.05; ANOVA with post-hoc Dunnett’s tests for each patient group vs. NL17), with the exception of PD DurIV in the derivation set and PD DurI in the validation set. An extended preclinical period of 10 to 15 years is suggested by the number of years from disease onset, estimated by the point on the fitted line of mean PDCore_subnet expression and mean duration for the PD I-V groups (p < 0.001; linear regression analysis), for which core expression was near zero. Expression values for 10 RBD subjects who are at risk for developing PD (see text) are depicted in cyan and displayed preclinically at the arbitrary x = -5 year mark. PDCore_subnet expression in this group was relatively elevated compared to the healthy subjects (p = 0.12; Student’s *t*-test). (B) Longitudinal Data: The increasing network expression with disease progression was more clearly evident in longitudinal data (Table S2) involving a group of 10 early stage PD patients (mean disease duration 2.6 y at baseline) (*top left*) tested at baseline and at four years of prospective follow-up and an independent group of 22 subjects at a later stage of disease duration (mean 11.7 y) (*top right*) tested at baseline and at one year follow-up. Significant increases in PDCore_subnet expression were seen between the baseline and final time point scores of each longitudinal sample (p = 0.0002 and p = 0.02, respectively; paired Student’s *t*-tests). Corresponding UPDRS motor ratings exhibited significant increases over time in the PD10 natural history cohort (*bottom left*) (p = 0.003; paired Student’s *t*-test). By contrast, motor ratings declined (i.e., improved) in the PD22 sham surgery cohort (*bottom right*) (p = 0.003) due to placebo effects. (For interpretation of the references to color in this figure legend, the reader is referred to the web version of this article.)

Based on the above, an invariant disease “common core subnet” was derived from the upper 5% of the bootstrapped PD subnet regional connections at wSparsity 85 ± 5% for the composite DurI-V samples (Fig. 6A), which involved 11 hub nodes (Table 2, column 6). A signed average core EC map vector (PDCore_net) matched to the polarity of the associated PC vector regions was used to calculate corresponding subject scores as inner products of the PDCore_net vector map with the individual subject voxel image vectors (see Methods). Group mean subject scores for the core network increased monotonically with mean disease duration, with the exception of DurIV, which presented positive rather than negative signed values for the caudate in the derivation PC (Fig. 1). Core Z-scored expression values for each PD group showed significant differences compared to the NL17 reference group (p = 0.01, 0.001, 0.003, 0.04, 0.0002 for DurI-V; Student’s *t*-tests) but was reduced in multiple comparisons (p = 0.17, 0.03, 0.002, 0.37, 0.0008; Dunnett’s test). Correlation of the PDCore_net region weights with the corresponding PD PC region weights was high in all but the fourth patient group (r = 0.945, 0.953, 0.973, 0.452, 0.955, p < 0.001; Pearson’s correlations) but was negative for NL17_PC3 regions (r = -0.7874, p < 0.001). Better discrimination of PD patients from healthy control subjects was obtained (p = 0.001, 0.0005, 0.002, 0.005, 0.001; Student’s *t*-tests) and for multiple comparisons (p = 0.05, 0.016, 0.001, 0.114, 0.0003; Dunnett’s test) by removing both caudate nuclei from the PDCore_net vector. To explore the potential of the resulting subnet as a disease biomarker, we plotted expression values for this core vector (termed PDCore_subnet) in the derivation set (Fig. 7A, *top*), revealing similar monotonic increases with advancing disease. Notably, region weights on PDCore_subnet exhibited strong correlations with corresponding PC weights in each of the PD groups including DurIV (r = 0.936, 0.946, 0.970, 0.971, 0.968, p < 0.001). Similarly, the PDCore_subnet region vector map correlates with the PDRP and PD33_PC1 vector maps (r = 0.945, and r = 0.992, respectively, p < 0.001) in the common region areas, thus mutually cross-validating these independently derived imaging features of PD. Correlation of mean PDCore_subnet Z-Scores with mean disease duration was significantly high (r = 0.98, p = 0.025) when group DurIV was excluded but marginal (r = 0.76, p = 0.16) with the DurIV group included. An estimated least squares regression line between the five mean expression values, extended negatively, predicted a mean presymptomatic phase of more than 10 years.

## 4 Validation of the common core subnet

To examine the prospective relevance of the PDCore_subnet, several patient data sets were utilized from our database (Supporting Demographics Table S1). An independent group of 69 PD patients, divided into groups of increasing mean disease duration similar to those of the derivation group, but for a wider age range [23–86 years], provided cross-validation indicating that group mean network expression of the common core subnet increases with mean disease duration even in patients with long disease duration (Fig. 7A, *bottom*), (r = 0.87, p = 0.05). Nevertheless, expression might not be as robust as it would be for separately derived phenotype-specific patterns, given that the common core subnet includes mainly *phenotype-invariant* elements. Prospective evaluation of expression score discrimination of PD patients from healthy control subjects was significant for all groups (p = 0.046, 0.003, 0.0002, 0.002, 0.008; Student’s *t*-tests) except for group DurI_69_ in multiple comparisons (p = 0.241, 0.015, 0.005, 0.005, 0.008; Dunnett’s tests). We additionally examined PDCore_subnet expression in 10 individuals with REM sleep behavior disturbance (RBD) (Supporting Demographics Table S2), who are at risk for developing PD in the future. While lacking clinical signs and symptoms of PD, these subjects exhibited PDCore_subnet expression at intermediate levels between healthy subjects and early PD DurI patients (Fig. 7A, Validation, *bottom*). Further, the common core subnet score and UPDRS motor ratings were evaluated for PD subjects in the derivation group (n = 70; Table 1, *last column*) and in the validation sample (n = 69; Table S2, *last column*). While in both sets of PD patients, PDCore_subnet scores and motor ratings increased with duration, consistent correlations between the clinical and subnet measures were not observed. Thus, the common core subnet is a disease-specific topographic feature of PD that is not directly associated with motor symptoms.

Given the limitations of cross-sectional comparisons of patient groups of different duration, we explored the changes in PDCore_subnet expression that occur over time in individual subjects. Indeed, increases were observed in longitudinal data (Supporting Demographics Table S2) from a group of 10 early stage PD patients (mean disease duration 2.6 years at baseline) (Fig. 7B, *top left*), retested at 2 years and 4 years (Huang et al., 2007) and from an independent group of 22 patients with longer disease duration (mean 11.7 years at baseline) (Fig. 7B, *top right*), scanned at baseline and at 6 and 12 months after receiving sham surgery as part of a randomized controlled study of gene therapy for advanced PD (Ko et al., 2014). Only the baseline and final time point measures are shown here to reflect the greatest differences for these relatively short time intervals. Significant increases in PDCore_subnet expression were seen between the baseline and final time point for each group (p = 0002 and p = 0.02, respectively; paired Student’s *t*-tests) although individual increases were at different rates. The PDCore_subnet is the same for each phenotype, but its expression in individuals differs because expression is a measure of similarity between the pattern nodal weights and the subject data. Mean values discriminated patients from the healthy NL17 control group (p = 0.0001; Student’s *t*-test) in both sets. Mean scores were generally in line with the predicted values of the common core duration timeline (Fig. 7A) and, as expected, were higher for the later stage PD22 group (Fig. 7B, *top right*) compared to the early stage group (Fig. 7, *top left*). Corresponding UPDRS measures (Fig. 7B, *bottom*) exhibited significant differences between time points (p = 0.003; paired Student’s *t*-test), but with notable declines in the PD22 group (Fig. 7B, *bottom right*). This suggests that while clinical UPDRS ratings were affected by sham effects (Ko et al., 2014), this was not the case for the PDCore_subnet scores. Thus, no significant correlation was observed between PDCore_subnet scores and UPDRS measures or between their respective time point differences.

## 5 Summary of procedure and findings

We performed an FDG PET data-driven joint analysis of multivariate regional spatial covariance using SSM/PCA and functional metabolic connectivity using GLASSO where no prior knowledge of disease network topography was assumed. Based on regional covariance analysis we identified specific PC patterns, for which pattern expression scores discriminated the disease group from matched healthy volunteer subjects for five different groups of age- and gender-matched patients at different stages of disease duration. The PCA analysis allowed us to identify the topographical patterns and relative weights of the component brain regions affected by the disease but does not reveal their underlying inter-regional associations. Using GLASSO on whole brain and disease specific PC partitions, the connectivity of brain areas was deduced from the inverse covariance of regional mean values, which allowed the most robust direct connections to be identified.

Based on the results of our entirely data driven approach, there are several network findings that characterize the disease in general; their clinical implications are discussed in the next section:

• Based on composite retrospective assessment, a common core subnet of interconnected regions that were affected by the disease but which were not duration- or phenotype-specific, is already present at the earliest stage of clinical manifestations (1–3 years duration).
• This common core subnet of at least 11 regions includes the left/right caudate, putamen, angular gyri, pons, right parietal inferior, right amygdala, and the vermis.
• Several regions of the common core subnet (notably the right caudate) act as global hubs connecting the disease network to external, primarily healthy, areas.
• The common core subnet is a feature of the disease that manifested at varying degrees of expression in over 95% of cross-sectional patient samples from 1 to 21 years of symptom duration.
• The earliest stages of disease duration involve higher relative metabolic activity concentrated in the putamen, pallidum, pons and amygdala than in other areas.
• Middle stages of disease duration are marked by the disconnection of common core subnet regions with frontal regions while connections of the metabolically negative caudate and parietal areas are widely expanded.
• Advanced stages manifest an extension of abnormal activity to previously minimally affected frontal areas involving dual involvement of the caudate nucleus in distinct disease networks that include DMN and PDCP regions.
• A hypothetical retrospective extension of the mean expression of the common core subnet suggested a prodromal phase of more than 10 years.
• The discriminative utility of the PDCore_subnet is essentially independent of standardized clinical disability measures in PD such as H&Y stage and UPDRS motor ratings.
• The findings suggest possible targets within the common core subnet for disease modification strategies, whereas deviant connections point to phenotype-specific treatment targets.

## 6 Discussion

The premise of this study has been to describe the overall network connectivity of progressive cross-sectional groups of PD patients of increasing disease duration, the differences in their prominent characteristics and how they evolve and relate to a hypothetical common core subnetwork. It is well accepted that the symptoms of PD and its progression are highly individual (Krüger et al., 2017, Nutt, 2016, Obeso et al., 2017, Selikhova et al., 2009). In this study, involving a large number of patients from early stages to advanced stages of disease progression, we examined whether despite broad variability across patients, stereotyped changes in metabolic connectivity can be discerned at the group level as the disease progresses. Although sparse whole brain representations allow the more prominent connections to be detected and analyzed, these may reflect healthy connections and/or aging effects that overshadow weaker disease-related connections at early disease stages or confound disease effects in later stages. As demonstrated in the current study using GLASSO/PCA, healthy connections such as those associated primarily with PC1 patterns that do not differentiate between PD and control subjects, remain robust – even in patients with long disease duration. By contrast, disease effects associated largely with PC2 patterns tend to increase at a slow rate with advancing symptoms. This is consistent with the generally slow rate of progression seen in this disorder (Krüger et al., 2017, Obeso et al., 2017).

### 6.1 Disease related group patterns

To elucidate disease-modulated connectivity, we focused on the partitions of the data attributed to specific disease-related PC patterns using our previously introduced methodology (Spetsieris et al., 2018, Spetsieris and Eidelberg, 2021) also described in Methods below. Although influenced by the disease stage of each derivation group, as well as other between-group factors, the derived PCs commonly exhibited stereotypical high positive nodal weights in the PD hallmark areas of the basal ganglia, thalamus, amygdala, pons and vermis and low values in parietal and occipital areas and caudate with one exception (Ko et al., 2018, Spetsieris et al., 2018, Spetsieris and Eidelberg, 2021). Notably, the expression of the most robust PD pattern in each group (as well as the pattern derived for the early DurI group) also discriminated patients from healthy subjects when applied to most of the other groups. In addition, these five patterns correlated with the established voxel-based discriminating pattern PDRP (r = 0.45, 0.67, 0.76, 0.56, 0.45, p < 0.001) and its regional counterpart PD33_PC1 (r = 0.36, 0.66, 0.74, 0.63, 0.70; p < 0.001; Pearson’s correlations) (Spetsieris and Eidelberg, 2021). Thus, PDRP accounted for at most 60% of the variance in the different PD patterns. This large difference may be expected considering that the PDRP is based on voxel-wise assessment, includes healthy subjects in its derivation, and involves a wide range of subjects of varying disease duration. The Dur patterns, by contrast, were limited to regional mean values for groups of subjects at specific intervals of disease duration. Nevertheless, these patterns performed similarly to previously characterized metabolic network biomarkers for PD (Schindlbeck and Eidelberg, 2018). The bootstrap displays depicted in Fig. 5 illustrate that the prominent regional components (nodes) of these patterns comprise elements of strongly connected subnetworks representing PD-specific node-to-node interactions associated with advancing motor and cognitive dysfunction as part of the disease process. In view of their differences, we sought to identify the common core elements of these networks that enabled them to function similarly in differentiating disease from subjects from healthy individuals in diverse other groups.

### 6.2 Early stage

In the earliest (DurI) group, robust regional hallmarks of motor dysfunction in the disease such as increased activity in the putamen and pallidum are obscured in the whole brain configuration that primarily reflects healthy connections of the first two PCs. They are evident, however, in the less prominent PC3 pattern subnet (Fig. 4, *bottom*). The evidence is even stronger in the bootstrap configurations in the joint ranges of 85 ± 5% and 92 ± 2% sparsity (Fig. 5, DurI). Indeed, the intense metabolic involvement of the putamen and pallidum is evident at the earliest clinical stages of the disease (Hoehn & Yahr stage I: (Tang et al., 2010a, Tang et al., 2020)) as a consequence of underlying nigral pathology (Braak stage III: (Braak et al., 2003, Hawkes et al., 2010, Schindlbeck and Eidelberg, 2018)). Thus, we conclude that comparatively greater activity in the basal ganglia and pons, as denoted by the positive weights of these regions relative to the other components of the disease network, is a distinguishing feature of the metabolic topography of PD, even at its early clinical stages. The regional changes seen in DurI_PC3 are in sharp contrast to the otherwise similar healthy pattern NL17_PC3 pattern from which it may have deviated. The healthy pattern’s topography (Fig. 4, *top right*, Fig. 5, *Healthy*) suggest that it is a non-parkinsonian network pathway.

### 6.3 Laplacian displays

Additionally, intense underlying clustering in DurI is evident in Laplacian based re-sorting of the DurI_PC3 subnet adjacency matrix (Fig. 3, *top right*) suggesting that the disease is already firmly rooted at the time of diagnosis. In the disease subnet from each Dur group, one or more high EC nodes behaved as hubs bridging the central clusters with low EC peripheral nodes. Several central nodes were identified as forming common clusters in all PD group subnets irrespective of symptom duration (Fig. 3 *right*, Table 2, magenta highlighted nodes). The presence of pronounced clustering in the common central hubs may reflect a compensatory mechanism or represent an intrinsic feature of the disease process (Ko et al., 2018, Koshimori et al., 2016). That said, the cohesiveness of the clusters in subnet space appears to diminish as the disease progresses (Fig. 3, *bottom right*). These findings suggest functional reorganization and spread of the disease to other network modules (Koshimori et al., 2016, Krüger et al., 2017).

### 6.4 Changes in progressive stages

From the earliest stage, there are signs of negative parietal activity in the angular gyri and the inferior parietal cortex that are apparent in all PD Dur groups (Fig. 5), although these negative areas gradually extend to other brain regions during the intermediate and late stages of the illness. These negative areas are associated with dysfunction in spatial orientation, perception and executive processes (Tahmasian et al., 2017, Wang et al., 2017). Increasing variability is seen as progressive neurodegeneration affects the overall integrity of connections (Fig. 3). With disease progression, additional networks become involved, as evident by elevations in DurIV_PC1 expression (Fig. S2) and increased clustering in global whole brain data (Fig. 3, *bottom left*).

### 6.5 Advanced stages

The advanced stage disease networks associated with PD DurIV and V contain elements of the novel metabolic DMN (Spetsieris et al., 2015), which may relate to neuropsychiatric symptoms such as hallucinations, depression, and attentional disturbances (Mohan et al., 2016, Yao et al., 2014), as well as cognitive dysfunction through involvement of PDCP regions (Schindlbeck and Eidelberg, 2018). However, the PDCP exhibited minor or no impairment for most patients. The primary PC1 patterns that are most associated with the DMN are non-discriminative in the first three Dur groups. However, DurIV_PC1 is partially discriminative in its derivation group and significantly discriminative in adjacent groups (Fig. S2D). Our observations regarding the advanced stage networks are compatible with the clinical observation that the transition to dementia in PD is typically quite gradual (Meireles and Massano, 2012).

### 6.6 Loss of frontal lobe connectivity

Interestingly, DurIV_PC1 is an asymmetric pattern with negative areas in the basal ganglia, parietal and occipital regions and relative positive activity in frontal and supplemental motor regions (Fig. S2A). The disconnected behavior of peripheral frontal areas that develops at late stage may be the basis for psychiatric symptoms such as psychosis, which occasionally accompany cognitive decline in these patients (Goldman, 2011, Nagano-Saito et al., 2004, Tekin and Cummings, 2002, Zahodne and Fernandez, 2008). This is also suggested by its partial topographic correlation with the normal metabolic DMN (r = 0.43, p < 0.001) (Mohan et al., 2016, Spetsieris et al., 2015, Yao et al., 2014) and with the healthy NL17_PC1 and PC2 (r = -0.47, -0.48, respectively, p < 0.001) (Fig. 4, *top left, middle*). These relationships point to prodromal loss of normal metabolic connectivity in the frontal lobe in advanced disease (Van Eimeren et al., 2009, Goldman, 2011, Nagano-Saito et al., 2004).

### 6.7 Common core subnetwork

Despite differences in the sparse disease subnets mapped for the various durations, we found evidence of a characteristic common “core” subnetwork (PDCore_net) that was present in all the PD Dur groups. This core comprised eleven highly interconnected nodes, with positive correlations between the basal ganglia, pons, and amygdala, and negative correlations with parietal association areas such as the angular gyrus. This accords with previous knowledge of the roles these nodes play in the PD disease process (Braak et al., 2003, Eidelberg, 2009, Ko et al., 2018, Tahmasian et al., 2017). The common core contains many regions receiving dopaminergic afferents (Hirano et al., 2008, Jourdain et al., 2016) and therefore may be particularly sensitive to dopamine agonist drug effects. The potential relevance of the PDCore_subnet to the design of new treatments lies in its ability to identify targets for general disease-modifying interventions for patients with prodromal/early stage disease.

### 6.8 Stage-dependent prominent regions

Other prominent regions, such as the pallidum, thalamus, amygdala, hippocampus and cerebellum are evident in multiple disease duration groups (Fig. 5) and in the composite representation of the first three groups I to III (Fig. 6B) but did not survive connectivity criteria for the composite subnet core (i.e., the upper 5% of connections for all five Dur groups) because of comparatively weaker connections or re-partitioning of connections among “normal” networks at the later disease stages (Fig. 5, Fig. 6A, B). The behavior of the amygdala and hippocampus is in keeping with the known association of these nodes with cognitive decline (Churchyard and Lees, 1997) as well as the involvement of the hippocampus in spatial learning (Schneider et al., 2017). Marked between-patient variability in this regard may be accompanied at later disease stages by topographical reorganization and/or fragmentation into separate PCs which do not individually reach significance.

### 6.9 Increasing expression of the common core subnet

By tracking changes in the expression of the PDCore_subnet across the PD Dur groups (Fig. 7A, *top*), we demonstrated linear increases in mean subject scores with longer disease duration, a relationship that was validated in an independent sample of 69 patients (Fig. 7A, Validation) and was further validated for individual subject increases in two independent longitudinal studies (Fig. 7B, *top*). These studies demonstrated that the PDCore_subnet expression is a reflection of general PD status, not significantly correlated with clinical motor ratings (Fig. 7B, *bottom*). As with the PDRP (Ko et al., 2014, Niethammer et al., 2018), longitudinal changes in the expression of the common core subnet are *not* affected by sham effects on clinical outcome measures determined under the blind. In both of the cross sectional PD samples, the x-axis intercept of the fitted lines relating PDCore_subnet expression to duration was approximately 10 or more years (Fig. 7A), in keeping with previous estimates of the preclinical period based on divergence from healthy aging patterns (Moeller and Eidelberg, 1997), as well as histopathological data (Hawkes et al., 2010).

### 6.10 Role of the caudate in PD

The deviant expression of the common core subnet in PD DurIV in the derivation data (Fig. 7A, *top*) was notably absent in the validation sample (Fig. 7A, *bottom*). We attribute this to connectivity changes associated with the change of the caudate’s function from a negative to a positive node in the derivation DurIV_PC2 pattern compared to PC2 in the other Dur groups. Although caudate connections were the most robust in both healthy and disease network bootstrap analysis (Fig. 6A, B, C, D), this nucleus was concurrently involved in multiple networks associated with motor as well as cognitive function (Alexander et al., 1986, Hirano, 2021, Holtbernd et al., 2015, Kish et al., 2008, Niethammer et al., 2013), which may influence its relative activity within the disease-specific networks in PD groups of differing duration. The contrasting positive and negative region weights on the caudate in the component PCs of PD DurIV may be related to disruption of connectivity in later disease stages (Fig. S2A, B) (Hirano, 2021, Niethammer et al., 2013). In addition, the DurIV_PC2 pattern is topographically related (r = 0.6) to the previously validated PDCP (Schindlbeck and Eidelberg, 2018), which also exhibits positive caudate weight (Fig. S2B). Indeed, prior studies have shown that dopaminergic projections to the caudate degenerate later in the disease course, coincident with elevation in PDCP expression levels (Huang et al., 2007, Mattis et al., 2016, Niethammer et al., 2013). The loss of caudate connectivity may be an indication that dopaminergic inputs to this region have declined sufficiently to result in cognitive impairment. The demonstrated increase in discriminative capacity of the core network subnet following omission of the nodal values for the left and right caudate on the PDCore_subnet vector (Fig. 7) suggests this region does not contribute to the discrimination of PD from healthy subjects given its involvement in normal as well as disease-related brain networks. The robust discriminative capacity of the PDRP (p = 0.0001, 0.0001, 0.0007, 0.0003, 0.0000002, Student’s *t*-tests for DurI to V, respectively, and p = 0.0067, 0.0039, 0.0001, 0.0213, 0.0000, post-hoc Dunnett’s test) may be in part due to the relatively smaller weights assigned to the caudate on this network, with a concomitantly greater difference in expression scores for PD vs. normal subjects for this pattern.

### 6.11 Role of the thalamus in PD

Similarly, the thalamus plays an important role in PD but does not facilitate discrimination between healthy subjects and PD patients because of its multifunctional role in both groups (Sommer, 2003). In our study, the left thalamus was prominent in most PD Dur group subnets (Fig. 5) and whole brain PD networks (Fig. 6C) but was also prominent in the healthy group subnet and whole brain network (Fig. 5, *Healthy*, Fig. 6D). Thus, common core elements may be targets for potential disease modification therapies, which are typically not directed at specific symptoms or clinical phenotypes. By contrast, regions associated with specific clinical manifestations (e.g., tremor, gait dysfunction) may be more suitable for symptomatic treatment. The multi-functional connectivity of the caudate, thalamus and other nodes delineated by our approach was a noteworthy observation that might be considered in future studies focusing on specific clinical subtypes. Indeed, the delineation of phenotypic differences in functional connections linking common core nodes to other regions may prove useful in the design of personalized therapies for PD symptoms.

## 7 Limitations

Various limitations may have affected the results of this analysis. The study was focused on determining common PD network hallmarks across phenotypes and the characterization of their progression. Thus, we did not perform individual or within-group analysis of changes over time in longitudinal PD cohorts. Nonetheless, the results were confirmed in longitudinal as well as cross-sectional datasets, showing the potential use of this methodology in mapping disease progression.

The regional parcellation scheme can have a significant effect on outcome (Fornito et al., 2010, Sporns, 2013). For instance, the cerebellum, apart from the vermis, was treated as two single left or right regions and thus, separate segments that may have specific motor or cognitive functionality have not been optimally assessed (Hacker et al., 2012, Jech et al., 2013).

The study focused on the most discriminative PC patterns although additional PC patterns may have exhibited limited discrimination in some groups. A composite multiple PC analysis for each group was not feasible in the context of this study. Although subjects were within the same age range in each group, the effect of the decrease in mean age of onset with advancing duration was not considered. Thus, the PD group with the longest disease duration was also the group with earliest onset, which may have underlying implications as to the specific disease phenotype (Chaudhary et al., 2018). The analysis is also influenced by the modest number of participants (17 in each group for DurI-III and HC) and fewer in later stages (DurIV, V). This is largely compensated by the high number of subjects in the composite groups and the robust connections derived from the precision matrices and bootstrap analysis.

Bootstrapping operations were limited to 100 iterations with viable solutions per group within each separate sparsity range and whole brain or subnet category. That said, results did not appreciably differ with respect to the number of iterations. Given the large composite computing demands of the employed algorithms, there was little practical advantage to increasing the number of iterations. In some subjects in each group, scores fell within the normal range. This was due possibly to disease variants or to overlapping networks associated with different phenotypes. Some PCs discriminated other patients from controls more significantly than patients in their own group. In cross-sectional data, specific clinical phenotypes may be more or less prevalent in groups other than that present in pattern derivation. Indeed, disease duration is not necessarily the only factor driving pattern to pattern variability. We acknowledge that the implementation of additional strategies could provide complementary information regarding the progressive transition from one stage to another of PD in general. Despite its limitations, this study helped identify an underlying common core network as a signature marker of the disorder.

## 8 Conclusion

Fundamental common network characteristics identify idiopathic PD as such within its wide spectrum of clinical disease expression. The joint methodology of Sparse Inverse Covariance Estimation using the Graphical Lasso in conjunction with SSM/PCA enabled the elucidation of prominent topological organization of functional metabolic connectivity in PD brain networks and its visualization in progressive stages of disease duration. The composite assessment of the prominent connections in disease-relevant PC subnetwork partitions of the data at different stages revealed a robust phenotype invariant common core PD subnet, for which mean expression increased with advancing disease. The core subnetwork was composed of regional elements and pathways that were readily familiar as PD-related in independent studies and further substantiated by their correlation with the established PDRP topography and by prospective validation in testing samples.

## 9 Methods

### 9.1 Data acquisition and software

Patients and healthy controls underwent fluorodeoxyglucose (^18^F-FDG) PET scans (GE Advance tomograph, Milwaukee, WI, USA) in the resting state at the Feinstein Institutes after overnight fasting to produce parametric maps of the distribution of glucose metabolism (considered to be a direct indicator of neural activity in the brain) (Meyer et al., 2017). Images were spatially normalized to a common Montreal Neurological Institute (MNI) stereotaxic space using SPM (https://www.fil.ion.ucl.ac.uk/spm). We performed the Scaled Subprofile Model of principal component analysis (SSM/PCA) using our MATLAB (Mathworks, Natick, MA)-based in-house Scan Analysis and Visualization Processor (ScAnVP) software (https://www.feinsteinneuroscience.org/). The methodology and pipeline of SSM/PCA was previously published (Spetsieris et al., 2013, Spetsieris and Eidelberg, 2011). Graphical analysis was performed using the Brain Connectivity Toolbox (BCT, brain-connectivity-toolbox.net) in conjunction with customized graph-theoretical ScAnVP-based algorithms and a customized implementation of the Graphical Lasso available on-line (graphicalLasso.m, by X. Chen, UIUC). Mean data values were obtained for 95 regions-of-interest (ROIs) defined in the automated anatomical labeling (AAL) atlas and additions as in Ko et al. (2018).

### 9.2 Group demographics

Five groups of PD patients (n = 17, 17, 17, 10, 9, respectively, for DurI to DurV) totaling 70 patients in the derivation group PD_70_ (Table 1), matched for age and gender, were defined based on symptom duration, ranging from 1–3 years in DurI to 17–21 years in DurV. To mitigate potential confounds due to age across the PD samples, we selected patients such that the range, mean, and standard deviation of this variable were similar for each duration subgroup. Indeed, significant correlations between age and duration were not observed in either the PD_70_ derivation group (r = 0.055, p = 0.65) or the PD_69_ validation sample (r = 0. 185, p = 0.13; Pearson's correlations). In these cross-sectional groups of PD patients, stages of disease duration did not necessarily correspond to symptom based stages of disease progression. A diagnosis of idiopathic PD was made in all patients in the derivation and validation groups; none exhibited evidence of dementia nor had clinical features of consistent with an atypical parkinsonian syndrome. Each of the PD duration subgroups (DurI-V) was compared to a group of age- and gender-matched healthy volunteer subjects (NL17). Additional subjects including 69 independent patients, similarly sorted based on symptom duration, were included in the validation group PD_69_ (Table S2). A previously studied reference group comprised of 33 PD patients (PD33; duration 5–18 years) (Ko et al., 2018, Spetsieris et al., 2015, Spetsieris et al., 2018), included 11 patients from the 70 subject derivation group (Table S2). PD33 is compared here in some segments because it is a patient group that was used along with matching controls to determine the PDRP, a major imaging biomarker of the disease (Eidelberg, 2009, Ma et al., 2007) and was previously studied with the same methodology used in this study (Spetsieris and Eidelberg, 2021). Here we studied a wider duration range in separate intervals to obtain a more concise representation of the signature cross-sectional connective core that characterizes all cases. The control group (NL17) was limited to the size of the Dur groups I to III (17 subjects) to avoid introducing variability due to differences in group size or differences in control groups since the control group was used to compare PC scores for each of the Dur groups separately. We attempted to match mean subject age and gender to reflect global PD cross-sectional demographic norms in all groups. In the two advanced Dur groups IV and V (10 and 9 subjects, respectively, combined duration 13 to 21 years), it was difficult to find enough patients to maintain this allocation. A small number of patients in the advanced validation groups included duration of up to 32 years. Two additional groups of 10 and 22 patients corresponding to early and later stages of disease duration for which longitudinal data were available from unrelated studies (Huang et al., 2007, Ko et al., 2014), were used to validate the increases in core subnet expression seen with disease progression.

Ethical permission was obtained from the Institutional Review Board of Northwell Health; written consent was obtained from each participant after detailed explanation of the procedures. The clinical and demographic characteristics of each group are provided in Table 1 and Table S2.

### 9.3 SSM/PCA analysis

With SSM/PCA multivariate spatial covariance analysis (Spetsieris and Eidelberg, 2011), voxel or regional mean data (D) are initially log transformed and then centered with respect to the subject and group mean values. This ensures that any multiplicative scalar factors are removed from the data prior to analysis. Thus, the topography of PCA metabolic networks reflects relative differences in metabolic values across regions, irrespective of global values (Dhawan et al., 2012, Spetsieris and Eidelberg, 2011). In multivariate analytical methods such as SSM/PCA, the influence of extraneous effects such as age can be minimized by selecting PCs with contrasting expression values in patients compared to closely matched healthy subjects. In mass univariate approaches such as SPM, by contrast, age and/or other subject descriptors can be directly entered into the model as nuisance variables. A complete set of orthogonal principal components **PC**_**k**_ and corresponding subject scores Score_jk_ are derived by performing PCA on the covariance data of each group’s data matrix so that any subject’s *j* data vector (**D_j_**) can be described as a sum of these PCs weighted by the subject scores (Eq. (1)):

(1) $$D_{j}=\Sigma_{k}Score_{\mathrm{jk}}\;{\mathrm{PC}}_{k}$$

The derived spatially overlapping orthogonal components **PC**_**k**_ may correspond to different data sources, including disease-related factors, other processes such as healthy aging, as well as extraneous effects such as, outliers, or noise. The PCs are unit normalized vectors of regional (or voxel) weights usually represented as Z-scored values. As is evident from Eq. (1), the partition (spatially overlapping layer) of the subject *j* data that is attributed to a specific principal component **PC**_**k**_ is equal to **PC**_**k**_ times the subject dependent Score_jk_ (Eq. (2)):

(2) $$D_{\mathrm{jk}}=Score_{\mathrm{jk}}\;{\mathrm{PC}}_{k}$$

The composite partitions of the group data over all subjects that are attributed to a specific PC are described here as the “subnet” data partition for that PC.

Disease-related components often described as disease networks or patterns can usually be found within the first few PCs representing major sources of variance thus reducing the group data to one or two specific PCs and their corresponding scalar subject scores (Eq. (3)):

(3) $$\mathrm{Scor}e_{\mathrm{jk}}=D_{j}^{T}\cdot {\mathrm{PC}}_{k}$$

The SSM/PCA analysis is performed separately for each of the Dur groups. Thus PCs derived in one Dur group do not necessarily correspond to the PCs derived in another group. We tested each set separately to determine the PC that best discriminated patients from controls judged by PC prospective expression scores.

In regional analysis, the PC vector region weights can be mapped to the corresponding three dimensional (3D) voxel space of the reference atlas regions to create individual pattern map representations (regional vector maps) that are visually comparable to voxel-based pattern representations. Prospective subjects can be tested by evaluating their expression scores as inner products of their regional data vector and the pre-derived PC pattern vector (Eq. (3)), referred to as topographic score ratings (TPR). Qualitatively comparable scores are obtained by using the image voxel data vectors and PC pattern regional vector maps (vector map TPR). We can test the probability that a particular PC originated from disease-related covariance sources using Student’s *t*-tests in comparisons of patient vs. control value scores for each group. In prospective multiple group comparisons of the discriminative capacity of a disease related PC against a fixed set of controls, we applied one-way ANOVA followed by the Dunnett’s test to evaluate p-values (Pokala, 2012). It is noteworthy that in SSM/PCA, voxel or regional data from the patients in a given disease group are usually combined with healthy control data to enhance variance, thus providing more robust and detailed patterns. However, graph theoretical analysis is regional and necessarily involves the evaluation of single, rather than combined, groups. Thus, to enable comparison of results from the two different methodologies, we applied regional PCA separately to the patient and control group data. Correlations between PC pattern vectors and graphical centrality regional vectors or maps were obtained by Pearson’s correlation.

### 9.4 GLASSO whole brain analysis

The GLASSO algorithm (Friedman et al., 2008) allows us to obtain a sparse representation of the inverse covariance matrix (precision matrix), even for modest samples, which reflects the prominent functional metabolic connections of the brain topology dependent on the level of sparsity. Here, sparsity refers to the degree of reduction of connections in the network. In accordance with the general concept of a sparse graph, we consider the sparsity of the associated matrix to increase as its density decreases, i.e., the number of zero entries increases with sparsity (De Luca, 2020). The elements of the precision matrix represent partial correlations, i.e., a non-zero element *ij* implies that *i* is directly connected to *j* (functionally correlated) in the absence of indirect influences from other regions. Zero elements imply that region *i* is conditionally independent of region *j*. The inverse covariance matrix Θ is estimated by maximizing the penalized Gaussian log-likelihood of the data using the empirical regional covariance matrix S (Eq. (4)). The level of sparsity is modulated by the regularization parameter ρ:

(4) $$\log\;\det\Theta -tr\left(S\Theta \right)-\rho \left|\left|\Theta \right|\right|1$$

where S = Empirical covariance matrix, Θ = Σ^-1^ = Inverse covariance matrix, tr denotes the matrix trace and ||Θ||1 is the L1 norm (the sum of the absolute values of the elements of Σ^-1^).

The magnitudes of the sparse inverse covariance elements are not considered reliable measures but are used to determine the 0/1 binarized adjacency matrix (A). Its non-zero elements determine the edges of regional nodes in its graphical representation. Using matrix A, we can visualize whole brain or subnetwork connectivity depending on whether data from the whole brain or the PC partition (PC subnet) are entered into the algorithm.

### 9.5 GLASSO/PCA subnet analysis

SICE reduces the connectivity to correspond to the most prominent connections but does not discriminate their source. In sparse representations, weaker disease-related connection may be obscured by normal networks, as well as other network connections that are minimally affected by the disease. Especially in initial disease stages, abnormal connections may not be prominent enough to be visualized by this method alone when applied to whole brain input. As in the previously introduced disease-focused joint analysis of the data from the historic PD33 reference group (Spetsieris et al., 2018, Spetsieris and Eidelberg, 2021), we apply the GLASSO algorithm to disease-specific PC partitions of the data, as well as to the whole brain data from each of the PD groups (DurI-DurV), and to the corresponding healthy group (NL17). Thus, for subnet analysis, the empirical covariance matrix S in Eq. (4) is the regional covariance of the PC partition data (specified by Eq. (2)) determined in each of the groups used for disease PC identification. Whole brain analysis is performed at or near maximum sparsity (defined below) that ensured a strongly connected graph involving all or most nodes (greater than 95%) in the whole data derivation samples. Partition data subnets derived within the specified whole brain maximal sparsity range do not necessarily involve all of the nodes of the full brain as the weakly connected nodes become disconnected. The algorithm determines the connected subnet of the data and evaluates its graphical parameters. A range interval of 85 ± 5% was considered as the maximal range that most likely involved strong brain connectivity in whole data bootstrap analysis. Within this 85 ± 5% range, partition subnet analysis revealed a smaller number of more densely connected regions corresponding to a lower network subnet sparsity. However, many of these nodes and edges are unreliable and eliminated in bootstrap analysis. Involvement of only the more reliable nodes and edges resulted in the effective sparsity levels closer to 85%. On the other hand, at 92 ± 2% whole brain sparsity, the whole brain data became disconnected, eliminating some of the disease-relevant nodes and edges – whereas the subnet connections in the original samples included these particular nodes and edges. However, in bootstrap analysis, further eliminations occur and the effective connected subnet sparsity, though greater, also falls closer to the 85% range. Thus, two whole brain range intervals (85 ± 5% and 92 ± 2%) were considered in bootstrap analysis; whole brain connectivity was evaluated at 85 ± 5% and partition subnets were evaluated separately within both ranges.

### 9.6 Graph theoretical analysis

Graph theoretical parameters for the whole brain and for subnet, sparse strongly connected adjacency matrices were evaluated using the BCT toolbox (https://www.brain-connectivity-toolbox.net/) as defined by Rubinov and Sporns (2010) and others. For the associated adjacency graph of strongly connected networks, every node is reachable from every other node. Because of the variable size of subnets resulting from the disconnection of weaker connections at high sparsity, normalized graphical values were evaluated. We examined degree and eigenvector centralities for different sparsity levels. Sparsity is defined here as the percent difference from the graph density (*d*) where *d* is the fraction of non-zero edges (*E*) to the number of possible edges of the undirected graph (*N(N-1)/2)* and N is the number of nodes (Eq. (5)):

(5) $$Sparsity\;=\;100\left(\left(1-d\right)\right),$$

where $d=\frac{2E}{N(N-1)},$ E is the number of edges and N is the number of nodes.

Preliminary values were obtained at the maximum sparsity that ensured a strongly connected graph involving all nodes in the whole data derivation (original) samples. In bootstrap data, the number of connected nodes varies. Sparsity levels were specified at 85 ± 5% in the whole brain analysis and at 85 ± 5% and 92 ± 2% in PC subnet analysis. Thus, in whole brain and bootstrap data, N is equal to 95 when it refers to the sparsity within the whole brain configuration (wSparsity) and N ≤ 95 when it refers only to the connected nodes that specify sparsity within the subnet space (nSparsity). In the text, sparsity refers to wSparsity values for both full brain and subnets unless otherwise stated. In this way, the measure can be used to determine a consistent range of values for the derivation of full brain networks and subnets with an equivalent numbers of edges. Within the sparsity range, 85 ± 5% to 92 ± 2%, a corresponding percent of possible connecting edges are excluded from the analysis and only 6% to 20% are included. For 95 ROIs the possible connections are 95 × 94/2, equal to 4,465, but only between 268 and 893 are processed.

It should be noted that graph theoretic parameters are only determined for the underlying connected network configuration whose value of nSparsity may be less than its value of wSparsity for a fixed number of edges. Further, in bootstrap analysis of prominent common connections, the network is further sparsified by the elimination of insignificant edges, resulting in group network configurations of prevalent nodes and edges with a higher “effective” sparsity.

A node’s eigenvector centrality (EC) is determined by the relative number of its important high-weighted node connections, thus reflecting its influence within the network (Lohmann et al., 2010). The EC vector is defined as the dominant eigenvector of a strongly connected adjacency matrix (Perron vector) and has strictly positive elements (Meyer, 2000). High EC nodes represent important graphical hubs. The EC vector node values of a graph typically correlate with the absolute value SSM PC vector region weights in order of importance. These values are therefore usually better correlated with the primary PC in whole brain sparse data (Ko et al., 2018) but relate more to the specific primary or secondary PCs when the graph network is derived from the PC’s subnet data (Spetsieris et al., 2018). Even higher correlation is obtained for EC vectors effectively signed similarly to the corresponding PC vector (Spetsieris and Eidelberg, 2021).

### 9.7 Graph Laplacian spectral decomposition

We further evaluated graphical nodal partitions of the adjacency matrix based on the nodal order of the Laplacian Fiedler vector (Fiedler, 1973, McDonald et al., 2012) in the whole brain and subnet space. The graph Laplacian matrix is evaluated as the diagonal degree matrix minus the adjacency matrix. Its Fiedler eigenvector corresponding to the second smallest eigenvalue was sorted by the magnitude of its elements. The ordered nodal index vector was used to similarly sort and redisplay the adjacency matrix (McDonald et al., 2012, Paulino et al., 1994) shedding light on the nodal partitioning of the whole brain image, and the topological structure of the PC subnetworks. The eigenvector centrality of the matrix and the polarity of the nodes in the corresponding disease PC was used to determine the color of the adjacency elements and help clarify the relationship of same and opposite polarity associations as well as the hub and lower degree nodal core/periphery configuration of each graph.

### 9.8 Bootstrap analysis

We performed bootstrap processing (Efron and Tibshirani, 1998) with replacement in over 100 resampled data case sets for each group in whole brain as well as in subnet analysis over a range of sparsity levels. Multiple internal iterations of the GLASSO algorithm at different penalty levels were performed to achieve convergence within each set (Friedman et al., 2008). Non-converging cases and networks that were outside the designated sparsity range were disregarded. More than 2000 cases were collectively assessed to determine 100 viable cases in each group for which the most robust connections were derived in progressive stages of PD and in healthy controls. As noted above, two range levels of sparsity (85 ± 5% and 92 ± 2%) were considered where sparsity refers to the value *wSparsity* evaluated within the full 95 ROI network for comparison of whole brain and subnetworks with a similar number of edges. 3D representations of prominent connections that occurred in all samples for each group and in composite assessment were derived for both bootstrap ranges in subnet data and whole brain data at the lower 85 ± 5% sparsity range. Graphical parameter values for brain networks at 92 ± 2% wSparsity are only evaluated for subnet configurations because strong connectivity was not attainable in the whole brain for most samples. Although at the level of wSparsity = 92 ± 2%, nSparsity is closer to 85% the high sparsity configurations do not consistently include all disease nodes of interest. Thus, for bootstrap samples a statistical evaluation is performed in both high and low sparsity ranges to determine the most prevalent edges and less significant edges are eliminated resulting in network configurations of prevalent nodes and edges with an “effective” network sparsity found to be within the 85% range.

### CRediT authorship contribution statement

**Phoebe G. Spetsieris:** Conceptualization, Methodology, Validation, Writing – review & editing, Software, Formal analysis, Writing – original draft, Visualization, Data curation. **David Eidelberg:** Conceptualization, Methodology, Validation, Writing – review & editing, Funding acquisition, Writing – original draft, Data curation, Supervision.

## Declaration of competing interest

The authors declare that they have no known competing financial interests or personal relationships that could have appeared to influence the work reported in this paper.

## Data Availability

Data will be made available on request.
