# Supplementary material for: Parkinson’s disease progression: Increasing expression of an invariant common core subnetwork
Source: Neuroimage Clin. 2023 Aug 22;39:103488. doi: 10.1016/j.nicl.2023.103488 (PMC10491857; doi:10.1016/j.nicl.2023.103488)
Supplement: Supplementary Data 1 [file mmc1.docx]

**Supplementary Material**

**Table S1. Subnetwork High Prevalence Bootstrap Connections**

| No. | Order | Prevalence | Atlas Index 1 | Atlas  Region 1 |  | Atlas Index 2 | Atlas  Region 2 |
| --- | --- | --- | --- | --- | --- | --- | --- |
| 1 | 1 | 500 | 72 | Caudate_R |  | 94 | Pons_L |
| 2 | 1 | 500 | 72 | Caudate_R |  | 95 | Pons_R |
| 3 | 1 | 500 | 71 | Caudate_L |  | 72 | Caudate_R |
| 4 | 1 | 500 | 71 | Caudate_L |  | 73 | Putamen_L |
| 5 | 1 | 500 | 72 | Caudate_R |  | 65 | Angular_L |
| 6 | 2 | 498 | 94 | Pons_L |  | 73 | Putamen_L |
| 7 | 2 | 498 | 71 | Caudate_L |  | 65 | Angular_L |
| 8 | 2 | 498 | 71 | Caudate_L |  | 74 | Putamen_R |
| 9 | 2 | 498 | 71 | Caudate_L |  | 62 | Parietal_Inf_R |
| 10 | 2 | 498 | 72 | Caudate_R |  | 93 | Vermis |
| 11 | 3 | 497 | 94 | Pons_L |  | 65 | Angular_L |
| 12 | 3 | 497 | 94 | Pons_L |  | 66 | Angular_R |
| 13 | 3 | 497 | 72 | Caudate_R |  | 74 | Putamen_R |
| 14 | 3 | 497 | 72 | Caudate_R |  | 66 | Angular_R |
| 15 | 3 | 497 | 73 | Putamen_L |  | 66 | Angular_R |
| 16 | 4 | 496 | 94 | Pons_L |  | 74 | Putamen_R |
| 17 | 4 | 496 | 94 | Pons_L |  | 62 | Parietal_Inf_R |
| 18 | 4 | 496 | 72 | Caudate_R |  | 42 | Amygdala_R |
| 19 | 4 | 496 | 71 | Caudate_L |  | 95 | Pons_R |
| 20 | 4 | 496 | 71 | Caudate_L |  | 94 | Pons_L |
| 21 | 5 | 495 | 72 | Caudate_R |  | 73 | Putamen_L |
| 22 | 5 | 495 | 71 | Caudate_L |  | 66 | Angular_R |
| 23 | 5 | 495 | 65 | Angular_L |  | 93 | Vermis |
| 24 | 6 | 494 | 94 | Pons_L |  | 93 | Vermis |
| 25 | 6 | 494 | 94 | Pons_L |  | 95 | Pons_R |
| 26 | 6 | 494 | 65 | Angular_L |  | 66 | Angular_R |
| 27 | 7 | 492 | 66 | Angular_R |  | 62 | Parietal_inf_R |
| 28 | 8 | 491 | 66 | Angular_R |  | 74 | Putamen_R |
| 29 | 9 | 490 | 72 | Caudate_R |  | 62 | Parietal_Inf_R |
| 30 | 9 | 490 | 65 | Angular_L |  | 62 | Parietal_Inf_R |
| 31 | 10 | 489 | 95 | Pons_R |  | 62 | Parietal_Inf_R |
| 32 | 11 | 487 | 95 | Pons_R |  | 65 | Angular_L |
| 33 | 12 | 485 | 71 | Caudate_L |  | 93 | Vermis |
| 34 | 12 | 485 | 74 | Putamen_R |  | 93 | Vermis |
| 35 | 13 | 484 | 66 | Angular_R |  | 93 | Vermis |
| 36 | 14 | 483 | 73 | Putamen_L |  | 93 | Vermis |
| 37 | 15 | 482 | 65 | Angular_L |  | 73 | Putamen_L |
| 38 | 16 | 478 | 74 | Putamen_R |  | 65 | Angular_L |

*High prevalence (weighted) edges in 500 composite bootstrap sample subnet adjacency matrices (100 samples in each group DurI to DurV) at wSparsity 85±5%. The 38 most common edges listed occurred in over 95% (478 or more) of the total sample adjacencies constituting less than 1% (0.0085) of the total number of 4465 possible undirected edges over the whole brain.*

**Table S2. Validation Group Demographics**

**Supplementary Figure Legends**

**Figure S1. Procedural Pipeline.** **(A,** *top***)** Original, group data for each PD duration subgroup (Dur I through V) and NL controls were analyzed using whole brain regional GLASSO (*left*) and SSM/PCA (*middle*). **(A,** *bottom***)** The GLASSO analysis was also performed in a single PC disease relevant partition layer of the same group sample overlapping the entire brain. **(B)** Bootstrap analysis was performed over multiple samples separately for the whole brain and for the selected partition layer overlapping the brain and was repeated independently for each of the groups. These bootstrap analyses were also repeated within two separate ranges of graph sparsity. **(C)** In composite assessment of 20 bootstrap analyses (5×2×2) for the 5 PD groups, we separately determined the number of connections that are significantly stable in whole brain and partition data. In addition, for the control group NL17, 4 analyses (2×2) were performed (in whole brain and for the discriminative PC, at the two separate sparsity ranges). **(D)** Graph theoretic analysis was performed for the resulting bootstrap significant network connections of each group and for the composite significant connections of the combined whole brain and partition groups separately. Prospective validation in independent data sets was performed for the significant core subnetwork of the composite partitions.

**Figure S2. Dur IV Dual Discriminating Partitions.** Orthogonal views of regional vector maps over an MRI background of the disease patterns PC1 and PC2 in group IV are presented in panels **(A)** and **(B)**. Both patterns showed significant differences in subject score values for pattern expression in different disease groups (*green rectangles*) compared to matched NL17 healthy subject scores using Student’s *t*-tests, and significant or borderline significant differences with Dunnett’s post-hoc correction for multiple comparisons **(D, E)**. DurIV_PC1 (**D**) was not discriminative for group DurIV but separated the two adjacent patient groups (DurIII and DurV) from control (NL17) values (p~0.05 and p~0.04, respectively, Dunnett’s tests); DurIV_PC2 (E) showed the greatest discrimination of group IV from healthy subjects (p~0.03, Student’s *t*-test), although this group difference did not survive correction for multiple comparisons (p=0.14, Dunnett’s test). DurIV_PC2 scores also discriminated group DurII from NL17 subjects (p~0.006, Student’s *t*-test), with a trend-level group difference after correction (p~0.08, Dunnett’s test). By comparison, pattern DurV_PC2 **(C)** appears to incorporate elements of both DurIV_PC1 and DurIV_PC2 but highly correlates with DurIV_PC1 (r=0.63, 0.15, respectively). Correlation of DurIV_PC2 (**B**) is high with PDCP (r=0.62), whereas correlation of DurIV_PC1 with PDCP was negative (r=-0.19). The DurIV_PC1 pattern (**A**) correlated strongly (r~0.43) with voxel weights of the DMN.
