# Supplementary material for: Parkinson’s disease progression: Increasing expression of an invariant common core subnetwork
Source: Neuroimage Clin. 2023 Aug 22;39:103488. doi: 10.1016/j.nicl.2023.103488 (PMC10491857; doi:10.1016/j.nicl.2023.103488)
Supplement: Supplementary Data 2 [file mmc2.pdf]

Fig. S1 A

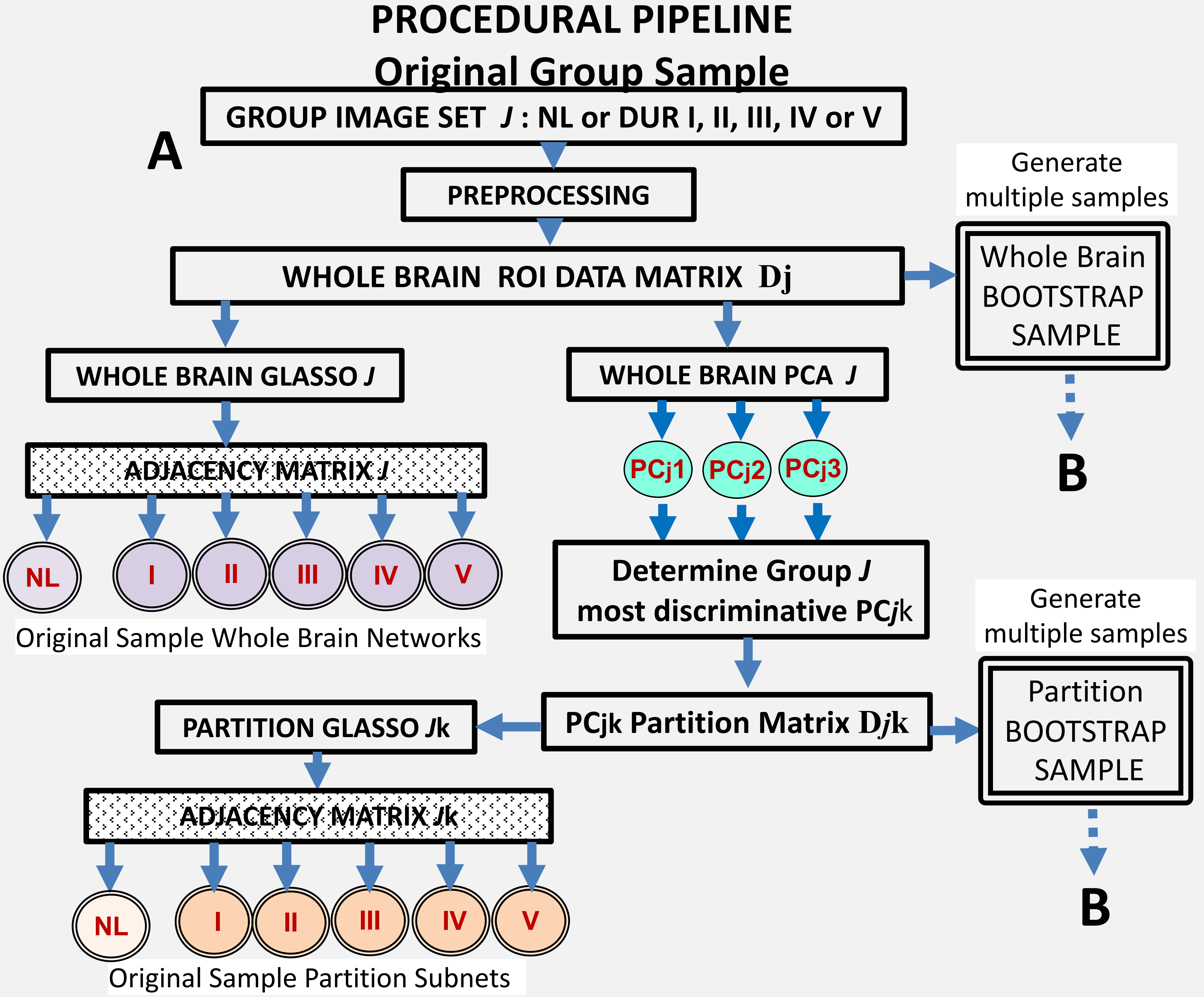
PROCEDURAL PIPELINE
Original Group Sample
A
GROUP IMAGE SET J : NL or DUR I, II, III, IV or V
PREPROCESSING
WHOLE BRAIN ROI DATA MATRIX Dj
Generate multiple samples
Whole Brain BOOTSTRAP SAMPLE
B
WHOLE BRAIN GLASSO J
WHOLE BRAIN PCA J
ADJACENCY MATRIX J
PCj1
PCj2
PCj3
NL
I
II
III
IV
V
Original Sample Whole Brain Networks
Determine Group J most discriminative PCjk
Generate multiple samples
Partition BOOTSTRAP SAMPLE
PARTITION GLASSO Jk
PCjk Partition Matrix Djk
ADJACENCY MATRIX Jk
B
NL
I
II
III
IV
V
Original Sample Partition Subnets

Fig.
S1
B,C,D

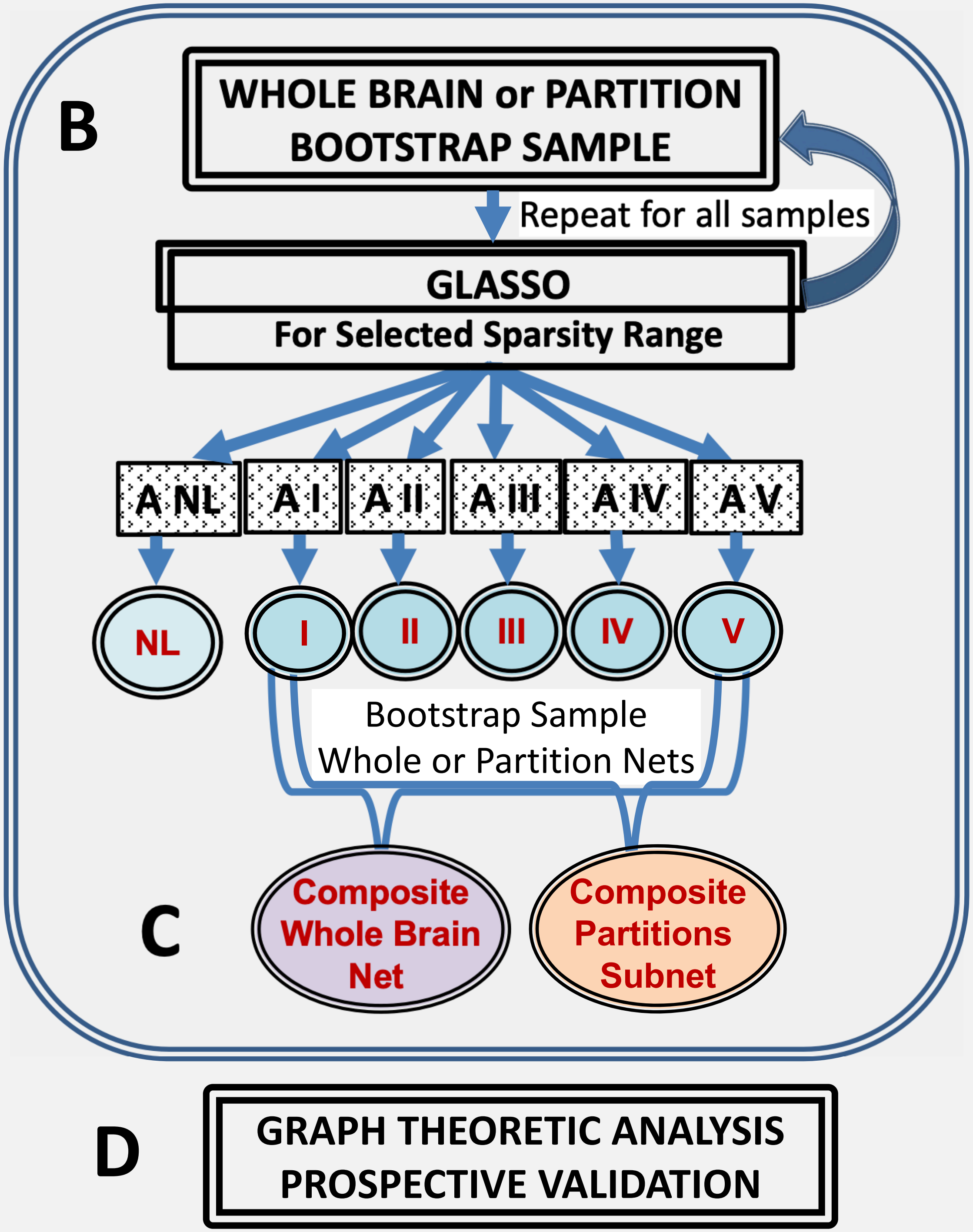
B
WHOLE BRAIN or PARTITION
BOOTSTRAP SAMPLE
Repeat for all samples
GLASSO
For Selected Sparsity Range
A NL
A I
A II
A III
A IV
A V
NL
I
II
III
IV
V
Bootstrap Sample
Whole or Partition Nets
C
Composite
Whole Brain
Net
Composite
Partitions
Subnet
D
GRAPH THEORETIC ANALYSIS
PROSPECTIVE VALIDATION

Fig. S2

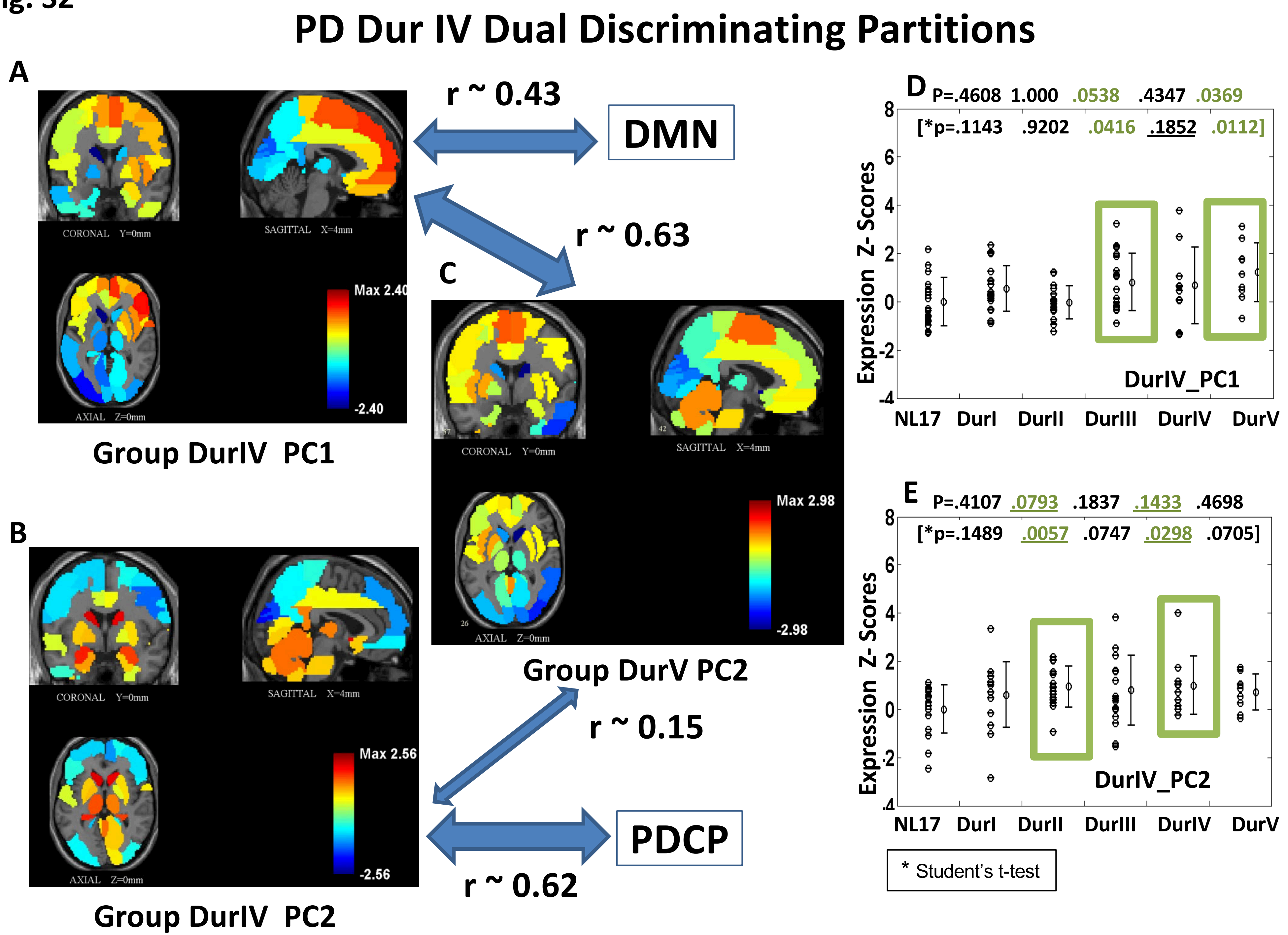

PD Dur IV Dual Discriminating Partitions
A
CORONAL Y=0mm
SAGITTAL X=4mm
AXIAL Z=0mm
Max 2.40
-2.40
Group DurIV PC1
r ~ 0.43
DMN
r ~ 0.63
C
CORONAL Y=0mm
SAGITTAL X=4mm
AXIAL Z=0mm
Max 2.98
-2.98
Group DurV PC2
r ~ 0.15
B
CORONAL Y=0mm
SAGITTAL X=4mm
AXIAL Z=0mm
Max 2.56
-2.56
Group DurIV PC2
r ~ 0.62
PDCP
D P=.4608 1.000 .0538 .4347 .0369
[*p=.1143 .9202 .0416 .1852 .0112]
Expression Z- Scores
DurIV_PC1
NL17 DurI DurII DurIII DurIV DurV
E P=.4107 .0793 .1837 .1433 .4698
[*p=.1489 .0057 .0747 .0298 .0705]
Expression Z- Scores
DurIV_PC2
NL17 DurI DurII DurIII DurIV DurV
* Student's t-test
